# Supplementary material for: Abnormal center of mass feedback responses during balance: A potential biomarker of falls in Parkinson’s disease
Source: PLoS One. 2021 May 27;16(5):e0252119. doi: 10.1371/journal.pone.0252119 (PMC8158870; doi:10.1371/journal.pone.0252119)
Supplement: S1 File — (PDF) [file pone.0252119.s001.pdf]

S1 File

Medications

Medication information was available for all participants with PD and 17/23 participants without PD. The majority (42/44) of PD patients were prescribed antiparkinsonian medications; the remaining two patients were early in the disease course and had not yet begun pharmacotherapy. Of those taking medications, 38/42 were on carbidopa-levodopa, either as conventional/extended release formulations or formulated as Stalevo or Rytary. Adjunctive agents included dopamine agonists (N=23), monoamine oxidase B inhibitors (N=14), entacapone (N=4), and other antiparkinsonian or anti-tremor agents including amantadine (N=5), trihexyphenidyl (N=1), apomorphine (N=1), the norepinephrine prodrug droxidopa (N=1), propranolol (N=3) and gabapentin (N=1). Two patients were taking the muscle relaxants cyclobenzaprine (N=1) and baclofen (N=1) for complications such as nocturnal cramps.

Some PD patients were taking the cholinesterase inhibitors donepezil (N=2) and memantine (N=3), and approximately one third (N=14) were taking psychiatric medications (SSRI, N=6; atypical antidepressants, N=4; SNRI, N=4, SARI, N=1). Among the Non-PD group for whom medication information was available, one person was prescribed bupropion; otherwise all neurotypical participants denied significant neurological or musculoskeletal problems. Common geriatric medications without psychiatric effects (statins, omeprazole) were frequently observed in both groups but were not abstracted.

Differences in peak muscle activity and CoM motion across groups

In addition to SRM analyses reported in the main text, gross magnitudes of CoM motion and normalized EMG magnitudes were compared between PD and Non-PD groups with independent *t*-tests (Table S1). Peak values were calculated 200, 400, and 675 ms after perturbation onset for absolute values of acceleration, velocity, and displacement, respectively in the anterior-posterior direction after averaging across replicates of each perturbation direction for each participant. Peak values for EMG were calculated during fixed windows 100-600 ms after perturbation onset.

Table S1. Comparison of gross CoM kinematics EMG activity between study groups.

Parameter	Young N=6	Non-PD N=18	PD N=44	P
Backward perturbations				
CoM kinematics				
Peak acceleration (g)	0.098±0.004	0.098±0.004	0.095±0.007	0.18
Peak velocity (cm/s)	11.9±0.5	11.3±2.5	12.3±0.9	0.07
Peak displacement (cm)	4.3±0.3	4.5±1.3	4.3±0.5	0.73
EMG activity, nu				
Peak TA, left	0.08±0.5	0.25±0.15	0.39±0.22	<0.01[*,††,‡]
Mean TA, left	0.03±0.01	0.08±0.04[a]	0.11±0.07	<0.01[*,††,‡]
Peak MG, left	0.71±0.09	0.80±0.11	0.80±0.08	0.08
Mean MG, left	0.21±0.04	0.29±0.09	0.27±0.07	0.12
Peak TA, right	0.09±0.05	0.28±0.17	0.36±0.17	<0.01[*,†]
Mean TA, right	0.03±0.01	0.10±0.07	0.13±0.07	<0.01[*,†]
Peak MG, right	0.70±0.11	0.80±0.07	0.79±0.11	0.17
Mean MG, right	0.22±0.05	0.29±0.07	0.27±0.08	0.13
Forward perturbations				
CoM kinematics				
Peak acceleration (g)	0.074±0.007	0.088±0.009	0.086±0.007	<0.01[*,††]
Peak velocity (cm/s)	11.6±0.5	11.2±2.2	12.1±1.1	0.10
Peak displacement (cm)	3.9±0.7	4.3±1.6	4.2±0.7	0.77
EMG activity, nu				
Peak TA, left	0.84±0.13	0.93±0.04	0.90±0.10	0.08
Mean TA, left	0.27±0.05	0.36±0.07	0.34±0.09	0.04[*,†]
Peak MG, left	0.33±0.18	0.37±0.13	0.32±0.16	0.51
Mean MG, left	0.10±0.05	0.14±0.06	0.12±0.06	0.25
Peak TA, right	0.84±0.09	0.90±0.05	0.88±0.08	0.28
Mean TA, right	0.27±0.08	0.34±0.07	0.34±0.08	0.14
Peak MG, right	0.39±0.31	0.33±0.15	0.40±0.23	0.47
Mean MG, right	0.12±0.11	0.12±0.05	0.14±0.07	0.48

Abbreviations: nu, normalized units; TA, tibialis anterior; MG, medial gastrocnemius. *Significant difference between groups, ANOVA. [†]P<0.05, [††]P<0.01; young vs. old, post hoc F-test, Holm-Bonferroni correction. [‡]P<0.05 PD vs. non-PD, post hoc F-test, Holm-Bonferroni correction. Results from the left leg are reported in the main text.

Recording and processing of kinetic, kinematic, and EMG data

All perturbation response data were collected and processed using procedures described previously.[1–4] Perturbations were applied using a custom perturbation platform (Factory Automation Systems, Atlanta, GA) driven by servo motors and controlled by industrial motion controllers. Platform acceleration was measured using a three-dimensional accelerometer directly mounted onto the platform (Analog Devices, Norwood, MA). Platform position was measured using a linear variable differential transformer (MTS Systems, Cary, NC). Ground reaction forces were captured using two six-axis commercial load cells directly mounted onto the platform (AMTI, Watertown, MA).

Analog data (platform kinematics, ground reaction forces, and EMG) were collected simultaneously and synchronized using Vicon (Oxford Metrics, Denver, CO) equipment at either 1080 or 1200 Hz depending on equipment version. Analog signals were anti-alias filtered with single pole hardware filters (500 Hz) prior to analog-to-digital conversion. Subsequent filtering was performed in software with third-order zero-lag Butterworth filters using *filtfilt.m* in Matlab. Platform signals were low-pass filtered at 30 Hz, and ground reaction forces were low-pass filtered at 100 Hz.

Kinematic marker data were collected at 120 Hz and synchronized to analog signals using Vicon hardware and software. Body segment kinematics were derived from a custom 25-marker set that included head–arms–trunk (HAT), thigh, shank, and foot segments (Vicon, Centennial, CO).[17] CoM displacement was calculated from kinematic data as a weighted sum of segmental masses.[83] CoM displacement was low-pass filtered (50 Hz) and numerically differentiated to derive the CoM velocity. CoM acceleration was then calculated as the difference between ground reaction force divided by subject mass and platform acceleration.

Surface EMG activity was collected from the following lower limb muscles: tibialis anterior (TA), medial gastrocnemius (MGAS), biceps femoris long head, rectus femoris, soleus, and vastus medialis. Only TA and MGAS were studied numerically. Silver/silver chloride disc electrodes were placed 2 cm apart at the motor point.[5] EMG data were recorded using

telemetered EMG (Konigsburg, Pasadena, CA, or Motion Lab Systems, Inc., Baton Rouge, LA) and processed offline (high-pass, 35 Hz, de-mean, rectify, low-pass, 40 Hz).[1–4]

Treatment of stepping responses

Perturbation trials that induced stepping responses or arm movement were excluded from analyses during initial processing in Vicon software. Stepping responses were identified programmatically by identifying trials in which the ground reaction force magnitude below either foot decreased below 20 N. Because of the data processing pipeline used, in which trials could be excluded at any of several stages of processing (initial processing in Vicon software via visual inspection, examination of recorded but unregistered data traces, statistical comparison of detailed numerical values), it was not feasible to systematically track exclusions. The number of trials excluded for any reason was ≈10%.

Preparation of kinematic signals for SRM Analysis

Anterior-posterior components of CoM motion were used in all analyses. We hypothesized that muscle spindles transiently encode acceleration when a muscle is stretched starting from rest, with the encoding ending abruptly as force and strain accumulate within the fiber. Although the mechanisms for such a "stiction" response are not known, it could be caused by the rapid detachment of cross-bridges.[6,7] We empirically modeled the muscle spindle stiction response by allowing acceleration encoding for a fixed time period τ after the onset of platform acceleration and deceleration. In an earlier study of young healthy individuals, values of τ were fixed at 75 ms.[3] Here, we fixed τ at 75 ms after initial perturbation acceleration and at 200 ms after initial perturbation deceleration based on preliminary investigations. During periods in which acceleration feedback was eliminated, acceleration signals were allowed to exponentially decay to zero with time constant 5 ms. This approach was taken because we reasoned that during these two periods, the agonist and antagonist muscles were lengthened sufficiently to detach cross-bridges, whereas during the middle of the perturbation response, lengthening would be insufficient.

SRM optimization

All optimizations were performed using the interior point algorithm implemented in *fmincon.m*, an implementation of that described in the literature.[8] Optimizations were performed in Matlab r2018b. In all cases, background activity levels identified from the start of recording to 50 ms after perturbation onset were removed from EMG recordings prior to SRM fitting. For balance-correcting feedback, a single optimization was performed to identify parameters k_a, k_v, k_d, and λ, with search bounds[9] described in Table S2.

For directionally nonspecific feedback, separate optimizations were initially performed to identify balance-correcting parameters k_a, k_v, k_d, and λ, and nonspecific parameters k_a', k_v', k_d', and λ'. In order to preserve initial burst peak values in reconstructions, limits on k_a' were set individually for each reconstructed EMG trace based on the ratio of peak EMG activity to peak acceleration. For each EMG trace, a parameter A was calculated as the ratio of the maximum value of normalized EMG activity calculated over a time window 150–275 ms after perturbation onset to the maximum value of the corresponding acceleration trace. The lower search bound for k_a' for that trace was then set to 90% of this value. A similar operation was employed to preserve peak values of braking activity 650-800 ms after perturbation onset by constraining k_a. After these two separate optimizations identified optimal values of parameter sets $(k_., \lambda)$ and $(k_.', \lambda')$, the feedback gains from the two parameter sets were concatenated into the initial guess for a final optimization. Lower and upper bounds for gain parameters during this search were set to ±10% of initial guess values; lower and upper bounds for delay parameters during this search were set to within ±10 ms of initial guess values. In all cases, additional parameters supplied to *fmincon.m* were as follows: *TolX,* $1e^{-9}$; *MaxFunEvals,* $1e^{5}$; *TolFun,* $1e^{-7}$. Remaining parameters were set to defaults. No manual tuning of optimization was performed.

Table S2. SRM parameter search values.

Parameter	Lower Bound	Initial Guess	Upper Bound
k_a' (nu/g)	0	10	15
k_v' (nu*s/cm)	0	0.01	0.04
k_d' (nu/cm)	0	0.01	0.04
λ' (ms)	90	140	210
k_a (nu/g)	0	10	15
k_v (nu*s/cm)	0	0.01	0.04
k_d (nu/cm)	0	0.01	0.04
λ (ms)	60	150	250

Comparison of identified SRM parameters across groups

Numerical values for identified SRM parameters for TA and MG are summarized in Tables S3 and S4, respectively. Independent omnibus and post-hoc F tests were applied to each parameter in each perturbation direction as described in the main text. Significant differences between groups for TA during backward perturbations are described in the main text. No statistically-significant differences between groups were identified for TA during forward perturbations or for MG in either perturbation direction.

Table S3. Identified SRM parameters for TA.

Parameter	Young N=6	Non-PD N=18	PD N=44
Backward perturbations			
$k_a'^{***,\dagger\dagger\dagger}$	0.8±0.4 (0.3-1.3)	1.5±0.9 (0.4-3.3)	3.0±1.8 (0.7-6.7)
k_v'	0.001±0.001 (0.000-0.004)	0.004±0.005 (0.000-0.021)	0.004±0.006 (0.000-0.035)
$k_d'^{\dagger\dagger\dagger}$	0.002±0.002 (0.000-0.005)	0.020±0.013 (0.000-0.037)	0.022±0.011 (0.001-0.037)
$\lambda'^{\dagger}$	136±28 (107-178)	189±27 (120-220)	181±18 (139-211)
$k_a^{\dagger\dagger\dagger}$	1.1±1.0 (0.3-2.9)	5.2±2.4 (1.1-10.8)	4.8±2.1 (1.0-9.1)
$k_v^{\dagger\dagger}$	0.006±0.003 (0.003-0.012)	0.011±0.007 (0.000-0.025)	0.013±0.005 (0.004-0.024)
k_d	0.014±0.007 (0.006-0.026)	0.014±0.008 (0.001-0.026)	0.018±0.008 (0.004-0.038)
$\lambda^{\dagger}$	151±13 (135-163)	129±19 (93-159)	130±20 (90-161)
Forward perturbations			
k_a	10.0±1.7 (8.0-12.0)	9.9±0.7 (8.6-11.4)	9.6±1.0 (7.0-12.4)
k_v	0.026±0.010 (0.011-0.038)	0.026±0.013 (0.000-0.040)	0.029±0.007 (0.008-0.040)
k_d	0.011±0.008 (0.000-0.020)	0.010±0.010 (0.000-0.028)	0.012±0.01 (0.000-0.040)
λ	126±12 (104-135)	144±16 (112-174)	134±12 (104-163)

Values are presented as sample mean ± sample standard deviation (sample range). ***Significant difference between Non-PD and PD groups, P<0.001. †††,††,†Significant difference young and older groups, P<0.001, P<0.01, P<0.05. Numerical values shown for backward perturbations are shown graphically in Figure 2C.

Table S4. Identified SRM parameters for MG.

Parameter	Young<br>N=6	Non-PD<br>N=18	PD<br>N=44
Forward perturbations			
k_a'	1.7±2.3 (0.0-6.1)	1.0±1.2 (0.0-4.9)	1.7±1.3 (0.0-5.2)
k_v'	0.005±0.008 (0.000-0.021)	0.004±0.006 (0.000-0.020)	0.004±0.005 (0.000-0.020)
k_d'	0.014±0.011 (0.004-0.029)	0.018±0.013 (0.000-0.037)	0.015±0.010 (0.000-0.040)
λ'	170±26 (143-196)	184±26 (129-220)	175±22 (126-216)
k_a	3.2±1.7 (1.0-6.2)	4.7±2.1 (1.9-9.4)	3.8±1.8 (0.8-9.7)
k_v	0.010±0.006 (0.005-0.020)	0.008±0.007 (0.001-0.025)	0.009±0.005 (0.000-0.025)
k_d	0.016±0.012 (0.004-0.031)	0.020±0.011 (0.000-0.020)	0.022±0.009 (0.005-0.038)
λ	126±33 (90-178)	135±18 (89-165)	132±24 (79-184)
Backward perturbations			
k_a	7.9±1.0 (6.8-9.6)	7.5±1.3 (5.9-10.6)	7.6±1.2 (4.3-10.6)
k_v	0.010±0.002 (0.008-0.013)	0.015±0.006 (0.005-0.025)	0.017±0.007 (0.004-0.032)
k_d	0.009±0.008 (0.001-0.026)	0.011±0.009 (0.000-0.032)	0.009±0.008 (0.000-0.028)
λ	130±6 (122-134)	142±24 (107-192)	144±16 (103-185)

Values are presented as sample mean ± sample standard deviation (sample range). No statistically-significant differences between groups were observed.

Goodness of fit

Goodness of fit between average SRM reconstructions and average recorded EMG traces was evaluated with VAF (variance accounted for) and R^2. VAF was defined as 100•the square of Pearson's uncentered correlation coefficient[10] and was calculated as in previous studies.[2,11,12] R^2 was calculated by built-in function *regress.m*. In cases in which one or the other criterion could not be calculated due to rank deficiency, values of 0 were imputed.

Differences in goodness of fit between groups for each muscle in each perturbation direction were assessed with separate ANOVAs. Significant initial F tests were followed with Holm-Bonferroni-adjusted post-hoc independent-samples *t*-tests comparing: 1) PD vs. Non-PD, and, 2) HYA vs. older (PD or Non-PD). Identified goodness of fit values are summarized in Table S5. Very few statistically-significant differences were identified: a small decrease in VAF (1%) was noted among young compared to older participants in TA during backward perturbations and a moderate decrease in R^2 (0.09) was noted among PD compared to Non-PD in MG during forward perturbations.

Table S5. Goodness of fit values.

Parameter	All	Young	Non-PD	PD
VAF, %				
Backward perturbations				
TA*	82±6	81±7†	82±6	82±5
MG	81±7	80±3	79±11	81±5
Forward perturbations				
TA*	77±11	71±5	71±17	79±6
MG	82±7	78±6	84±5	82±7
R^2				
Backward perturbations				
TA	0.60±0.11	0.50±0.06	0.64±0.11	0.59±0.11
MG	0.53±0.12	0.52±0.05	0.49±0.17	0.54±0.11
Forward perturbations				
TA	0.63±0.13	0.59±0.08	0.59±0.19	0.65±0.09
MG*	0.44±0.13	0.38±0.09	0.51±0.12	0.42±0.13‡

Values are presented as sample mean ± sample standard deviation. *P<0.05 between groups, ANOVA. †P<0.05 between older and younger groups, ‡P<0.05 between PD and Non-PD groups, post-hoc tests.

Changes in reconstructed muscle activity after the addition of diffuse sensorimotor feedback

In order to quantify changes in fits to recorded EMG data associated with additional nonspecific CoM feedback channels in the SRM, we compared adjusted R^2 values (R^2_a) and peak reconstructed EMG levels 100-600 ms after perturbation onset before and after the addition of the new channels. These analyses were conducted using only data from the PD group, and only using data in which muscles were initially shortened by perturbations (backward for TA and forward for MG), in the interest of parsimony.

R_a^2, referred to as the adjusted coefficient of determination,[10] was calculated from R^2 values calculated by built-in function *regress.m* according to the formula:

$$R_a^2 = 1 - \frac{n-1}{n-m-1}(1-R^2)$$

where m indicates the number of explanatory variables and n indicates the number of data points. Values were calculated assuming $m = 4$ independent explanatory variables for the balance-correcting feedback pathway, $m = 8$ independent explanatory variables for the balance-correcting and nonspecific feedback pathways, and $n = 1620$ data points in all cases.

We assessed differences in R_a^2 after the addition of nonspecific feedback with paired t-tests. The addition of nonspecific feedback was associated with highly statistically-significant ($P<<0.001$) increases in R_a^2 for both TA during backward perturbations and MG during forward perturbations. Average increases in R_a^2 were 0.18±0.15 (range, -0.03–0.66) for TA, and 0.13±0.14 (range, -0.03–0.56) for MG.

We assessed differences in reconstructed peak muscle activity with a paired t-test for each muscle and compared the increases between TA and MG with an independent samples t-test. After the addition of additional nonspecific CoM feedback channels, reconstructed peak muscle activity 100-600 ms after perturbation onset increased significantly for both muscles ($P<0.001$). Average increases in peak muscle activity were 0.23±0.11 normalized units (range, 0.08–0.43) for TA, and 0.14±0.10 normalized units (range, 0.03–0.40), for MG. Increases in peak muscle activity were significantly higher in TA ($P<0.001$).

Associations between PD and falls

Simple associations between PD and fall history

Participants were classified as having 0, 1, or ⩾2 falls in the 6 months prior to study enrollment as described in the main text. Fall history in each group is summarized in Table S6. Simple associations between the presence of PD and fall history were assessed with chi-squared tests of homogeneity. Previous falls were significantly more prevalent among the PD group (47% vs. 12%; P=0.008; Figure 3A). Similar results were obtained if young participants were excluded from this analysis (47% vs. 17%; P=0.032).

Table S6. Retrospective 6-month fall history stratified by group.

Falls in prior 6 months	Young or Non-PD N=24	PD N=43
≥2	0 (0)	12 (28)
1	3 (12)	8 (19)
0	21 (88)	23 (53)

Values are presented as n (%).

Associations between SRM parameters and falls

Associations between SRM parameters and falls were assessed in two ways. First, we used multivariate ANOVAs to assess variation in SRM parameters with fall classification (0, 1, or ⩾2 falls in the prior 6 months, as described above). Second, in order to assess the strength of associations between SRM parameters and falls, we used negative binomial regression to assess associations between SRM parameters and the number of falls over the prior 6 months. In the subset of participants for whom fall frequencies were available, it ranged from 0 to 1 (Non-PD) or 180 (PD).

Primary analyses considered TA SRM parameter k_a'. For completeness, secondary analyses were performed in which the analysis was iterated for each of the remaining parameters identified during backward perturbations and shown in Figure 2. Family-wise false positive rate across these analyses was controlled with a Holm-Bonferroni sequential procedure.

Associations between SRM parameters and fall classification

Values for each identified parameter were analyzed with ANOVA with a factor for fall history classification (0, 1, or ⩾2 falls in the prior 6 months), as well as clinical and demographic covariates known to be associated with fall risk: age, female sex, presence of PD, and presence of FOG.[13,14] PD patients were classified as freezers if they scored > 1 on FOG-Q[15] item 3, indicating freezing more than once per week.[16] The age variable was transformed to z-scores prior to entry into analysis assuming mean value 68 y and standard deviation 7 y.

Compared to among participants with no fall history, k_a' was significantly increased (115%, P<0.001, ANOVA; Figure 3B) among those with ⩾2 falls in the prior 6 months after controlling for age, sex, presence of PD, and presence of FOG. In this multivariate analysis, k_a' was significantly increased among participants with PD (37%, P=0.042; Figure 3C) and tended to increase with age (≈8%/decade, P=0.116). No significant effects of sex (P=0.742) or presence of FOG (P=0.584) were identified. No significant differences were identified in k_a' between participants with 1 and 0 falls over the prior 6 months.

Overall, results were very similar when the analysis was iterated with young healthy participants excluded. The highly significant effect of fall history (111%, P<0.001) was retained, the effect of PD (37%, P=0.051) was weakened slightly, and the effect of age (≈10%/decade, P=0.348) was slightly increased in magnitude.

We applied identical analyses to other SRM parameters in order to identify potential associations with fall history. Significant associations were identified for TA k_v' (P=0.010), TA k_d' (P=0.024), and for TA k_a (P=0.032). However, none remained statistically-significant after correction for N=11 simultaneous tests using a Holm-Bonferroni procedure.

Associations between SRM parameters and fall number

In a subset of participants (N=22, PD; N=11, Non-PD), the number of falls over the 6 months preceding study enrollment was available for analysis. Because we noted anecdotally that PD patients with extreme values of k_a' also often reported very frequent falls, we evaluated whether this association held at the group level using negative binomial regression. We fit the following equation:

$$log\ log\ (\hat{f}) = \beta_0 + \beta_{ka'} k_a' + \beta_{age} age + \beta_{female} female + \beta_{PD} PD + \beta_{FOG} FOG$$

where $\hat{f}$ is the estimated number of falls for a given patient, which is assumed to follow a negative binomial discrete probability distribution. The regression parameters $\beta_.$ describe the intercept (β_0), and slope parameters for each of the continuous variables k_a' and age, and for each of the three dichotomous indicator variables for the presence of female sex, PD, and FOG. The age variable was standardized according to mean 68 y and standard deviation 7 y prior to entry into analysis.

The use of a negative binomial distribution was strongly supported by overdispersion in reported fall frequencies, with mean 6.9 falls/6 months and variance 875.8 falls/6 months (range 0–180). By group, sample means (variance) were 0.1 (0.1) and 12.2 (1517.7) in Non-PD and PD, respectively.

Negative binomial regression demonstrated that k_a' was highly significantly associated with increased fall frequency (P<0.001, Figure 3D). Identified regression parameters are shown in Table S7. Associations between fall frequency and k_a' remained highly statistically significant (P<0.001) when the analysis was iterated with young participants excluded.

Table S7. Negative binomial regression model parameters describing association between number of falls and SRM parameter k_a'.

Parameter	Estimate	95% CI	P Value
Intercept	-3.49	-5.49, -1.50	<0.001
k_a'	0.66	0.32, 1.00	<0.001
PD	1.24	-0.93, 3.40	0.264
Age	0.41	-0.39, 1.22	0.317
Female	1.31	-0.08, 2.70	0.064
FOG	1.07	-0.46, 2.60	0.171

Abbreviations: FOG, freezing of gait.

Within-subject variability of SRM parameters

We calculated test-retest reliability in a convenience sample of N=6 PD patients (age, 68±4 y; disease duration, 5±4 y; MDS-UPDRS-III score, 36±10) for whom repeat testing results were available ≈12 and ≈16 weeks after initial enrollment. The average time between observations was 33±5 days (range, 28-42 days), over which one would presumably expect little change in CoM control. We calculated intraclass correlation coefficient ICC (2,1) following the methodology of Shrout and Fleiss.[17]

The identified value of ICC (2,1) was 0.94 (95% CI 0.60-0.99), which is considered "excellent" according to cutoff values proposed by Koo and Li.[18] Average within-subject variability (CV) was 9.5%.

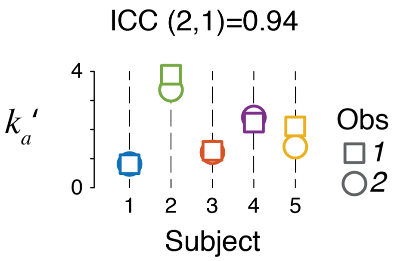

Figure S1. Within-subject variability of SRM parameter $k_a{}'$.

Associations between SRM parameters and clinical and demographic variables after controlling for age

In the main text, associations between $k_a{}'$ and candidate clinical variables were summarized with Pearson product-moment correlation coefficients and classified as negligible or non-negligible according to criteria proposed by Cohen.[19] In order to control for the impact of age on identified values of $k_a{}'$, we iterated correlational analyses with and without age included as a covariate and assessed the resulting changes in correlation coefficients.

In unadjusted analyses, non-negligible correlations were identified between $k_a{}'$ and age, PD duration, LED, MDS-UPDRS-III total score, MDS-UPDRS-III Postural Stability score, Mini-BESTest reactive postural control score, and MoCA score, with more impaired values of $k_a{}'$ associated with worse clinical measures in all cases.

In analyses adjusted for effects of age, on average correlations were attenuated in magnitude by 22 ± 29%. Only associations with PD duration, LED, and Mini-BESTest reactive postural control score remained non-negligible in adjusted models (Table S8). In these cases, more abnormal values of $k_a{}'$ were associated with longer PD duration, higher amounts of pharmacotherapy, and more impaired reactive postural control.

Table S8. Single and multiple regression results describing associations between clinical variables and SRM parameter k_a'.

Variable	r	95% CI	P Value	
Univariate associations				
Age	0.18†	-0.13, 0.49	0.24	
PD duration	0.26†	-0.04, 0.56	0.09	
LED	0.19†	-0.13, 0.50	0.23	
MDS-UPDRS-III	0.10†	-0.21, 0.41	0.52	
MDS-UPDRS-III PIGD	0.04	-0.27, 0.35	0.79	
MDS-UPDRS-III Postural Stability	0.10†	-0.21, 0.41	0.50	
MDS-UPDRS-III Tremor	0.08	-0.23, 0.39	0.60	
Mini-BESTest	-0.02	-0.38, 0.35	0.93	
Mini-BESTest Reactive Postural Control	-0.12†	-0.48, 0.24	0.49	
Mini-BESTest Dynamic Gait	0.07	-0.29, 0.44	0.69	
Mini-BESTest Sensory Orientation	0.07	-0.30, 0.43	0.71	
Mini-BESTest Anticipatory Balance	-0.05	-0.42, 0.31	0.76	
MoCA	-0.12†	-0.43, 0.19	0.44	
Associations adjusted for age				Change from unadjusted
PD duration	0.23†	-0.09, 0.54	0.18	-12%
LED	0.20†	-0.11, 0.51	0.22	+5%
MDS-UPDRS-III	0.05	-0.27, 0.38	0.48	-50%
MDS-UPDRS-III PIGD	0.03	-0.28, 0.34	0.50	-25%
MDS-UPDRS-III Postural Stability	0.08	-0.24, 0.39	0.45	-20%
MDS-UPDRS-III Tremor	0.07	-0.24, 0.38	0.46	-13%
Mini-BESTest	0.00	-0.39, 0.39	0.95	-100%
Mini-BESTest Reactive Postural Control	-0.11†	-0.51, 0.28	0.78	-8%
Mini-BESTest Dynamic Gait	0.07	-0.30, 0.45	0.87	0%
Mini-BESTest Sensory Orientation	0.07	-0.30, 0.45	0.87	0%
Mini-BESTest Anticipatory Balance	-0.04	-0.44, 0.36	0.93	-20%
MoCA	-0.09	-0.40, 0.23	0.44	-25%

Abbreviations: LED, levodopa equivalent dose. †Non-negligible effect.

Examples of antagonist activity in MG

Taken together with the results earlier studies examining muscle activation throughout the leg during balance, we interpret the absence of a statistical effect of the presence of PD on MG antagonist activity as evidence that the effect of PD on MG activity is substantially more variable than the effect of PD on TA activity, rather than as evidence that PD does not affect MG activity. We were somewhat surprised that we did not find that antagonist activity in MG was abnormal in PD at the group level in this sample, given that we have previously reported excessive MG antagonist activity during balance tasks using a similar paradigm in PD patients in the ON medication state.[20] However, in a subsequent extensive examination of the activation of 6 muscles throughout the leg performed on a subset of these data, we found that the presence of PD was associated with elevated antagonist activity across generally all muscles examined, but that when muscles were considered in isolation, the effect was statistically significant only in TA. Here, although some patients exhibited strong antagonist activity in MG (see Figure S2, upper panel), others exhibited very little antagonist activity (see Figure S2, lower panel). We therefore consider the most likely explanation for these results to be that PD likely affects MG activity, although in a manner that is substantially more variable than the effect of PD on TA activity.

Why the pathophysiological processes of PD might exert a more consistent effect on TA than on MG is unknown. Many potential mechanisms could explain this type of asymmetry, although there is scarce data in humans to establish a convincing case for any particular one. Heightened rigidity in flexor rather than extensor muscles in PD – particularly in early disease stages [21] – has been noted previously, and illustrates that PD disease processes can certainly exert differential effects on particular muscle groups. In particular, Denny-Brown[22] cited the “preponderance [of rigidity] in the flexor groups” as a potential explanation for the characteristic flexed abnormal posture seen in all the limbs in terminal PD. Others, while agreeing that flexors and extensors are affected in a differential manner by PD, argue that flexed postures result primarily from extensor impairment, rather than flexor rigidity, during voluntary movements,[23] with extensor impairment potentially contributing to reduced overall strength.[24] In general, because the representations of extensor and flexor muscles are

organized in rough topographic fashion within the basal ganglia and motor cortices [25] it is reasonable that different muscle groups could be affected at in different amounts as neurodegeneration progresses along the neuraxis,[26] although the precise mechanisms of this progression remain unknown.

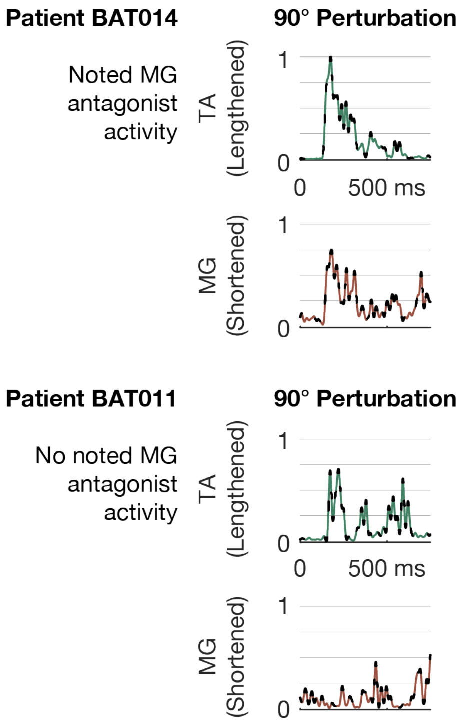

Figure S2. Examples of recorded MG antagonist activity in two participants, selected *post hoc* as exemplars of the range of MG antagonist activity seen among PD patients. Traces are averages of 3-6 replicates of 90° perturbations that initially lengthen TA and shorten MG. Muscle activity is normalized as described in the main text so that 1 corresponds to the maximum average value observed during perturbations in which the muscle is lengthened.

Additional studies in a larger number of patients with a larger number of muscles would be required to comprehensively assess the extent to which PD affects the sensorimotor control of flexors or extensors during balance, particularly because the sampling of ankle extensors is substantially more variable across studies than the sampling of ankle flexors. Here, for consistency with previous studies using the SRM approach in young healthy individuals, we considered TA and MG. However, while previous studies using perturbation paradigms to assess

balance in PD have almost uniformly recorded TA, the sampling of ankle extensors has been much more variable, precluding comparing results across studies. Of three earlier studies with methodological similarities to the testing paradigm here, the first recorded MG, but only considered backward perturbations of the support surface, so the antagonist activity of MG was never observed.[27] The others considered perturbation directions throughout the horizontal plane, but reported soleus (SOL) rather than MG.[28,29]

Simple association between SRM parameters and PD duration

A scatterplot of SRM parameter $k_a{}'$ vs. PD duration is presented in Figure S3. Values are adjusted for linear effects of age. Separate best-fit regression lines are presented for PD participants ⩾5 years duration and <5 years duration. Participants with higher values of $k_a{}'$ tend to be beyond 5 years PD duration. Not all participants with PD exhibit high values of $k_a{}'$, but all participants with high values of k_a' have PD. There is evidence from visual inspection that the relationship between $k_a{}'$ and PD duration has a nonlinearity at approximately 5 years, with a stronger and steeper relationship in the earlier period.

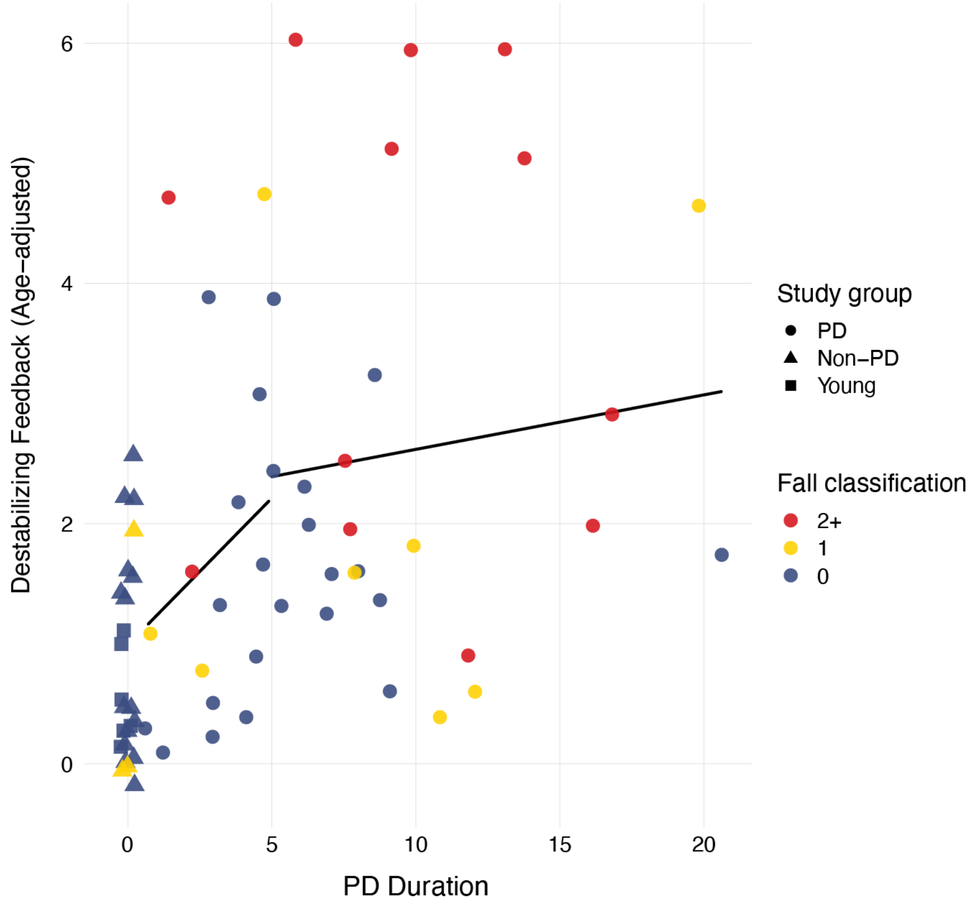

Figure S3. Comparison of $k_a{}'$ and PD duration. Separate best-fit lines are presented for duration < and ⩾5 years. A small amount of horizontal jitter has been added to aid in visualization.

Simple association between SRM parameters and freezing of gait

In primary analyses of associations between k_a' and falls, presence of freezing of gait (FOG) was controlled for as a dichotomized variable, as in previous studies.[13,16] The choice of using a dichotomized measurement for FOG in primary analyses was due to the limited validity of self-reported instruments for capturing moderate or small changes in FOG severity.[30,31]

In order to more fully examine potential associations between k_a' and FOG, we performed a secondary analysis post-hoc on the dataset of k_a' and an estimate of FOG severity, FOG-Q total score. These data were available for N=42 PD patients. Overall, Pearson's associations were non-negligible, *r*=0.18 (P=0.25) and *r*=0.14 (P=0.37) before and after adjustment for age, respectively.

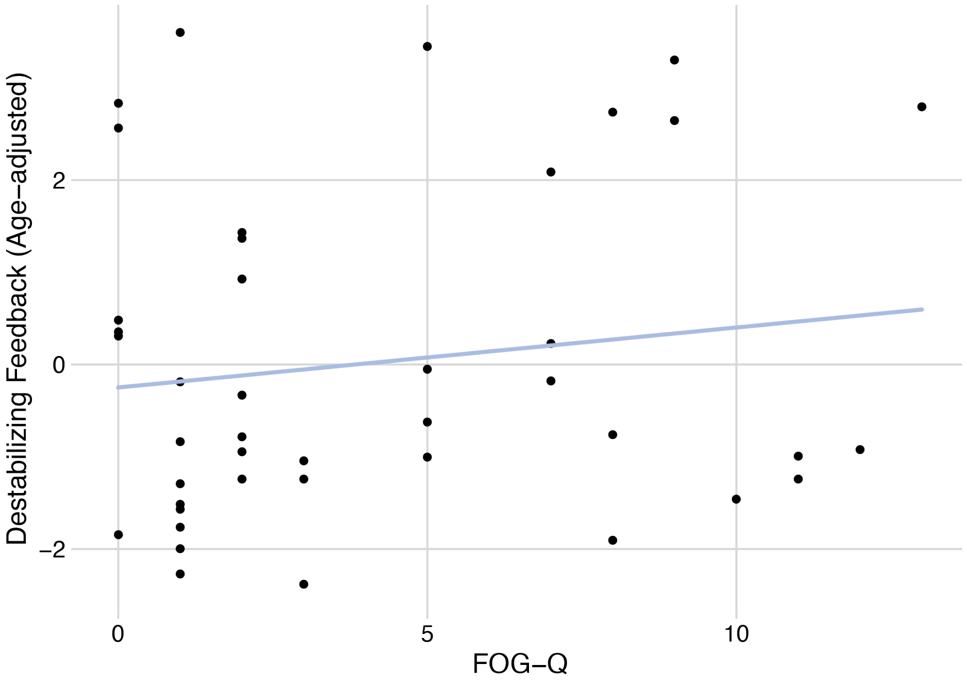

Figure S4. Comparison of k_a' (adjusted for age) and FOG-Q score.

Variation of SRM with PD phenotype

Based on earlier analyses of overall muscle activity levels [1] we did not expect large differences between PD patients with TD vs. PIGD vs. Indeterminate. We verified this with a one-way ANOVA (PIGD vs. TD vs. Indeterminate phenotype) applied post-hoc that identified no variation in average k_a' level across groups (P=0.94).

References

1. Lang KC, Hackney ME, Ting LH, McKay JL. Antagonist muscle activity during reactive balance responses is elevated in Parkinson's disease and in balance impairment. Franzén E, editor. PLOS ONE. 2019;14: e0211137. doi:10.1371/journal.pone.0211137

2. Safavynia SA, Ting LH. Long-latency muscle activity reflects continuous, delayed sensorimotor feedback of task-level and not joint-level error. J Neurophysiol. 2013;110: 1278–90. doi:10.1152/jn.00609.2012

3. Welch TDJ, Ting LH. A Feedback Model Explains the Differential Scaling of Human Postural Responses to Perturbation Acceleration and Velocity. J Neurophysiol. 2009;101: 3294–3309.

4. Welch TD, Ting LH. Mechanisms of motor adaptation in reactive balance control. PLoS One. 2014;9: e96440. doi:10.1371/journal.pone.0096440

5. Basmajian JV, Blumenstein R. Electrode Placement in EMG Biofeedback. Baltimore/London: Williams & Wilkins; 1980.

6. Getz EB, Cooke R, Lehman SL. Phase transition in force during ramp stretches of skeletal muscle. Biophys J. 1998;75: 2971–83.

7. Henatsch HD. [Pro and contra on the acceleration sensitivity of muscle spindles]. Bull Schweiz Akad Med Wiss. 1971;27: 266–81.

8. Waltz RA, Morales JL, Nocedal J, Orban D. An interior algorithm for nonlinear optimization that combines line search and trust region steps. Math Program. 2006;107: 391–408. doi:10.1007/s10107-004-0560-5

9. Welch TDJ. A feedback model for the evaluation of the adaptive changes to temporal muscle activation patterns following postural disturbance. Ph.D., Emory University and Georgia Tech. 2008.

10. Zar J. Biostatistical Analysis. Saddle River, NJ: Prentice-Hall; 1999.

11. Torres-Oviedo G, Macpherson JM, Ting LH. Muscle synergy organization is robust across a variety of postural perturbations. J Neurophysiol. 2006;96: 1530–1546.

12. Chvatal SA, Macpherson JM, Torres-Oviedo G, Ting LH. Absence of postural muscle synergies for balance after spinal cord transection. J Neurophysiol. 2013;110: 1301–10. doi:10.1152/jn.00038.2013

13. McKay JL, Hackney ME, Factor SA, Ting LH. Lower Limb Rigidity Is Associated with Frequent Falls in Parkinson's Disease. Mov Disord Clin Pr. 2019;6: 446–451. doi:10.1002/mdc3.12784

14. Paul SS, Canning CG, Sherrington C, Lord SR, Close JC, Fung VS. Three simple clinical tests to accurately predict falls in people with Parkinson's disease. Mov Disord. 2013;28: 655–62. doi:10.1002/mds.25404

15. Giladi N, Shabtai H, Simon ES, Biran S, Tal J, Korczyn AD. Construction of freezing of gait questionnaire for patients with Parkinsonism. Park Relat D. 2000;6: 165–170. doi:10.1016/s1353-8020(99)00062-0

16. McKay JL, Lang KC, Ting LH, Hackney ME. Impaired set shifting is associated with previous falls in individuals with and without Parkinson's disease. Gait Posture. 2018;62: 220–226. doi:10.1016/j.gaitpost.2018.02.027

17. Shrout PE, Fleiss JL. Intraclass correlations: uses in assessing rater reliability. Psychol Bull. 1979;86: 420–8.

18. Koo TK, Li MY. A Guideline of Selecting and Reporting Intraclass Correlation Coefficients for Reliability Research. J Chiropr Med. 2016;15: 155–63. doi:10.1016/j.jcm.2016.02.012

19. Cohen J. A power primer. Psychol Bull. 1992;112: 155–9.

20. McKay JL, Ting LH, Hackney ME. Balance, body motion, and muscle activity after high-volume short-term dance-based rehabilitation in persons with Parkinson disease: a pilot study. J Neurol Phys Ther. 2016;40: 257–68. doi:10.1097/NPT.0000000000000150

21. Rodriguez-Oroz MC, Jahanshahi M, Krack P, Litvan I, Macias R, Bezard E, et al. Initial clinical manifestations of Parkinson's disease: features and pathophysiological mechanisms. Lancet Neurol. 2009;8: 1128–39. doi:10.1016/S1474-4422(09)70293-5

22. Denny-Brown D. Diseases of the basal ganglia. Their relation to disorders of movement. Lancet. 1960;2: 1099–105.

23. Robichaud JA, Pfann KD, Comella CL, Brandabur M, Corcos DM. Greater impairment of extension movements as compared to flexion movements in Parkinson's disease. Exp Brain Res. 2004;156: 240–54. doi:10.1007/s00221-003-1782-0

24. Inkster LM, Eng JJ, MacIntyre DL, Stoessl AJ. Leg muscle strength is reduced in Parkinson's disease and relates to the ability to rise from a chair. Mov Disord. 2003;18: 157–62. doi:10.1002/mds.10299

25. Nambu A. Somatotopic organization of the primate Basal Ganglia. Front Neuroanat. 2011;5: 26. doi:10.3389/fnana.2011.00026

26. Braak H, Tredici KD, Rüb U, de Vos RAI, Jansen Steur ENH, Braak E. Staging of brain pathology related to sporadic Parkinson's disease. Neurobiol Aging. 2003;24: 197–211. doi:10.1016/s0197-4580(02)00065-9

27. Horak FB, Frank J, Nutt J. Effects of dopamine on postural control in parkinsonian subjects: scaling, set, and tone. J Neurophysiol. 1996;75: 2380–2396.

28. Dimitrova D, Horak FB, Nutt JG. Postural Muscle Responses to Multidirectional Translations in Patients With Parkinson's Disease. J Neurophysiol. 2004;91: 489–501. doi:10.1152/jn.00094.2003

29. Carpenter MG, Allum JH, Honegger F, Adkin AL, Bloem BR. Postural abnormalities to multidirectional stance perturbations in Parkinson's disease. J Neurol Neurosurg Psychiatry. 2004;75: 1245–54.

30. Hulzinga F, Nieuwboer A, Dijkstra BW, Mancini M, Strouwen C, Bloem BR, et al. The New Freezing of Gait Questionnaire: Unsuitable as an Outcome in Clinical Trials? Mov Disord Clin Pract. 2020; mdc3.12893. doi:10.1002/mdc3.12893

31. Snijders AH, Haaxma CA, Hagen YJ, Munneke M, Bloem BR. Freezer or non-freezer: Clinical assessment of freezing of gait. Parkinsonism Relat Disord. 2012;18: 149–154. doi:10.1016/j.parkreldis.2011.09.006
