# Supplementary material for: Abnormal center of mass feedback responses during balance: A potential biomarker of falls in Parkinson’s disease
Source: PLoS One. 2021 May 27;16(5):e0252119. doi: 10.1371/journal.pone.0252119 (PMC8158870; doi:10.1371/journal.pone.0252119)
Supplement: Attachment — Submitted filename: response-to-reviews.pdf [file pone.0252119.s002.pdf]

Response to Reviewers

We thank the editors and reviewers for their time and helpful comments. We are pleased that the reviewers found no substantial methodological concerns in our cross-sectional study to identify a novel neuromechanical biomarker of Parkinson's disease – and, further, of the frequency of falls among people with Parkinson's disease – that demonstrates excellent test-retest reliability. We have fully addressed the concerns of the reviewers for additional material in the discussion and limitations sections. We have prepared a point-by-point summary of reviewer comments and corresponding changes to the manuscript, which follows. Changes to the manuscript in response to these concerns are indicated by ***blue text***.

Here, we briefly summarize our response to the primary critiques that reviewers cited as reducing the overall impact of the findings, bringing into question the appropriateness of our balance testing paradigm and statistical analysis:

1) There was some concern amongst reviewers that our behavioral testing was not robust enough to predict falls because it did not cause participants to lose balance during the testing. However, we believe that the novelty of the result is that our analysis at a single balance perturbation level is associated with falls history, even though participants did not lose their balance during the testing. Because we are examining the underlying neurophysiological signals, we are able to detect muscle activity patterns that differentiate individuals with PD and those with PD who fall. We consider this analogous to a cardiac stress test, where electrophysiological signals can indicate cardiac health during a challenge that does not cause an adverse event.

2) A reviewer questioned our use of a logarithmic scale as being "misleading" – but this is a common approach when using negative binomial regression, a statistical technique common in epidemiological and clinical studies when the dependent variable exhibits substantial overdispersion. This is certainly the case with fall frequency, which is distributed with a very heavy tail given the large number of falls (>300/year) experienced by some patients.

It is difficult to overstate the importance of understanding, predicting, and preventing falls to people affected by PD, stakeholders, and clinicians. To highlight the significance and novelty of this result and its direct relevance to falls in people with PD we have revised the title to include the phrase "a new biomarker of falls":

Abnormal center of mass control during balance: a new biomarker of falls in people with Parkinson's disease

The point-by-point response to reviewer comments follows.

> **<u>Decision Letter, April 17, 2020</u>**

Dear Dr. McKay,

Thank you for submitting your work entitled "Abnormal center of mass control during balance is associated with falls in Parkinson's disease" for consideration by eLife. Your article has been reviewed by 3 peer reviewers, and the evaluation has been overseen by a Reviewing Editor and a Senior Editor. The following individuals involved in review of your submission have agreed to reveal their identity: John Jeka (Reviewer #2); Carolin Curtze (Reviewer #3).

Our decision has been reached after consultation between the reviewers. Based on these discussions and the individual reviews below, we regret to inform you that your work will not be considered further for publication in eLife.

Overall, the reviewers remarked that the findings of your manuscript were interesting, and noted that they did extend the argument for excessive muscle co-activation in Parkinson's disease. However, there were concerns about the model being mainly descriptive (and failing to include predictions of behavior). Additionally, one reviewer wondered about the significance of the findings given that the observed changes in EMG were associated with maintained stability (i.e., normal CoM). While we are not inviting a resubmission given that these, along with other issues, led the reviewers to question the impact value of the work, they also recognized that their opinion might change with substantial modifications addressing the range of concerns. Thus, you may consider if the paper could be reworked into a new submission. We would likely engage at least one new reviewer to look at the paper with fresh eyes.

*** Portable peer review ***
We would be happy to transfer the peer reviews, along with the reviewers' names (where they agree), to another journal of your choice. For example, in the last 12 months, we have facilitated the transfer of review material to a range of journals, including Biology Open, BMC Biology, EMBO Reports, eNeuro, the PLOS journals, the Journal of Cell Biology, Journal of Neuroscience, and the Journal of General Physiology. Such transfers are only undertaken in response to an explicit request by the corresponding author. For neuroscience submissions, please note that we participate within the Neuroscience Peer Review Consortium (http://nprc.incf.org/index.php/about/information-for-authors/).

Although the sharing of peer reviews can potentially help to save time, the editors of the receiving journal may decide to consult new referees. In addition, not all reviewers provide us with permission to share their identity with another journal. If you would like the eLife reviews to be taken into account during submission to another journal, please submit your article directly using their online manuscript tracking system.

eLife is highly selective, which means that the majority of submissions are rejected, but we thank you for sending your work for review and we hope you will submit to eLife again in the future.

Best wishes,

Nicole Swann

Reviewing Editor, eLife

Richard Ivry
Senior Editor, eLife

We thank the editors and reviewers for their time and helpful comments. We feel that we have addressed them, and that the manuscript is improved as a result. We have selected to transfer the revised manuscript for consideration in PLoS Computational Biology.

Reviewer #1 (General assessment and major comments (Required)):

This is a well-written manuscript that will be of interest to the movement disorders and motor control communities. In this study, the authors identify an unusual response of the tibialis anterior (TA) in persons with Parkinson's disease (PD) during recovery from a balance perturbation. They expand a previously published model to provide a mathematical control framework for understanding this response.

My comments and suggestions are included below:

1) I find it difficult to interpret the relevance of the modeling results given that there were no differences in the COM kinematics during the perturbation responses in persons with PD vs. non-PD. I understand that, within the proposed framework, this means that changes in TA activity could not be due to changes in movement and instead must be driven by changes in the model's parameter for sensitivity to acceleration. However, it is unclear how this change in sensitivity would result in instability given that there does not appear to be instability (from a COM perspective) in response to the perturbations. If the COM kinematic response to a perturbation does not change as a result of increased TA sensitivity, why would increased TA sensitivity to backward perturbations result in increased fall frequency?

Indeed the crux of our finding is that, despite similar CoM kinematics in response to perturbation, the activation of muscles differ in those with PD vs. non-PD, and even more so in those with PD who have a history of falls. Our findings indicate that the nervous system activates the incorrect muscle to a higher degree in PD fallers. The over-excitation of the antagonist muscle means that the agonist muscle must overcome not only the destabilizing forces due to the perturbations and gravity, but also the forces of the antagonist (TA) muscle. Our analysis further reveals the nature of the excessive TA antagonist activity to be a hyper-excited response to CoM acceleration, represented as k_a'. In the tested perturbation level, the agonist muscle was successful in overcoming both of these forces.

We believe that it is reasonable to measure impaired balance performance without inducing actual falls, if neurophysiological variables are measured. Similarly, one does not need to introduce actual atrial fibrillation during a stress test to measure impaired cardiac function. We hypothesize that that the excessive TA antagonist activity is an indicator of fall risk because it limits the effective torque that can be produce by the agonist muscles to restore balance (Sohn, McKay, and Ting, *Journal of Biomechanics* 2013; Simpson, Sohn, Allen, and Ting, *Journal of Biomechanics* 2015), which is only evident behaviorally when that limit is reached.

Consistent with this idea, we predict that those with low k_a' would be successful in maintaining balance at a higher perturbation level, whereas those with high k_a' would be more likely to fail at a higher perturbation level. This is a testable prediction that we will investigate in future studies. The strong relationship identified between ka' and retrospective fall frequency supports this approach.

We have now added the following text to the Results section immediately following the callout to Figure 1A, which depicts the perturbation paradigm:

To assess the balance control system without inducing falls, the perturbation parameters were refined over a series of studies in Parkinson's patients in both the OFF and ON medication states to be very near, but to not exceed, the level at which they could maintain balance without stepping. [1-4]

> **Perhaps a clearer insight would emerge if only persons with PIGD and/or impaired COM kinematic responses to the perturbations were included?**

We thank the reviewer for the interesting suggestion. We have now completed an analysis that compared k_a' values across PIGD, tremor-dominant, and indeterminate PD phenotypes and showed no differences between PIGD and the other groups (*P=0.94*).

These results support our prior finding that overall antagonist activation during perturbation responses is abnormally elevated in people with PD independent of phenotype (Lang, Hackney, Ting, and McKay, *PLoS ONE* 2019).

We have added the following material to the Results:

Among the PD group, there was no statistically-significant variation in k_a' across PD phenotypes (P=0.94, ANOVA on PIGD vs. TD vs. Indeterminate).

We have included the following material to the Supplemental Information:

Based on earlier analyses of overall muscle activity levels[9] we did not expect large differences between PD patients with TD vs. PIGD vs. Indeterminate. We verified this with a one-way ANOVA (PIGD vs. TD vs. Indeterminate phenotype) applied post-hoc that identified no variation in average k_a' level across groups (P=0.94).

> **2) It is also difficult to interpret how robust the relationship between the acceleration sensitivity parameter (ka') and fall frequency may be. The relationship in the PD+FOG group appears to be driven primarily by one participant who experienced > 100 falls in the past six months while no other participant in the group experienced > 5. Similarly, in the PD group, one participant experienced > 50 falls and no others > ~10. The y-axis scale makes Fig 3C somewhat misleading.**

We thank the reviewer for the comment. We disagree that the logarithmic y-axis scale in Fig 3C is misleading. The analysis depicted in Figure 3C is a negative binomial regression, which is

relatively common in epidemiological studies in which the outcome of interest is characterized by a non-Gaussian distribution with a "long tail."

We agree with the reviewer that there is pronounced overdispersion in the reported fall frequencies, in which the variance far exceeds the mean. This is very characteristic of fall frequencies reported by people with Parkinson's disease, which often are either very rare (once or twice a month) or very frequent (once or twice a day). In our data, the mean was 6.9 falls/6 months and the variance was 875.8 falls/6 months (range 0–180). By group, sample means (variance) were 0.1 (0.1) and 12.2 (1517.7) in Non-PD and PD, respectively.

The negative binomial regression process is designed specifically for data with this dispersion pattern, in order to ensure that, for example, the datum of the participant who experienced > 100 falls in the past six months does not outweigh the contributions of the other data, and has been recommended in the literature numerous times specifically for fall frequency data:

> Ullah, Shahid, Caroline F. Finch, and Lesley Day. "Statistical Modelling for Falls Count Data." *Accident Analysis & Prevention* 42, no. 2 (2010): 384–92. https://doi.org/10.1016/j.aap.2009.08.018.
>
> Gill, Dawn P., Guang Yong Zou, Gareth R. Jones, and Mark Speechley. "Comparison of Regression Models for the Analysis of Fall Risk Factors in Older Veterans." *Annals of Epidemiology* 19, no. 8 (2009): 523–30. https://doi.org/10.1016/j.annepidem.2009.03.012.
>
> Simpson, Lisa A., William C. Miller, and Janice J. Eng. "Effect of Stroke on Fall Rate, Location and Predictors: A Prospective Comparison of Older Adults with and without Stroke." *PLoS ONE* 6, no. 4 (2011): e19431. https://doi.org/10.1371/journal.pone.0019431.

To address this concern, we have added the above references to the manuscript text as follows:

Negative binomial regression is common in epidemiological studies and has been frequently used for fall frequency data.[38–40]

> **Relationships between all other clinical data and ka' are relatively weak.**

We acknowledge that the relationships between clinical variables and ka' are "small" or negligible according to cutpoints established by Cohen (1992). This was largely expected because the other clinical variables have generally poor ability to predict fall risk (Duncan, 2012; Bloem, 2001). However, regardless of the strength of the associations identified, we believe that these results are important to include as they are of primary interest to clinician scientists from neurology and physical therapy, among other fields. To address this concern, we have added the following material to the Results:

Given the sample size and limited emphasis on reactive postural control in the MDS-UPDRS-III – and to a lesser extent, Mini-BESTest, both of which are considered inadequate for assessing fall risk[41] – we did not expect to identify strong correlations with clinical variables.

> **3) The temporal dynamics of the muscle activity are only qualitatively described. The authors focus primarily on the amplitude of the responses but, at least in the individual data shown in Figs 1B and 2A, it appears as though the antagonist TA response is delayed in time relative to the balance-correcting MG response.**

We respectfully disagree with the reviewer, as our SRM analysis explicitly reconstructs the temporal dynamics of muscle activity using a linear combinations of CoM kinematics. To our knowledge, we have developed the most precise method of quantifying muscle activation patterns in reactive balance control by formulating the timecourse of activity as a feedback controller. In identifying the model parameters, we are not simply examining the amplitude of muscle activity per se, but rather it's amplitude and timecourse relative to the sensory input of the perturbation, characterizing the sensitivity of motor output to sensory input. As such, the temporal dynamics are quantitatively described in the section of the results: *"Antagonist CoM acceleration feedback delay times are consistent with long-loop activity,"* in the subsection of the methods: *"Delay differences between stabilizing and destabilizing pathways,"* and in Figure 5. The corresponding portion of the results reads:

"Identified SRM delay parameters in each of the identified pathways were not different in PD versus non-PD groups (p=0.54). The TA-TA stabilizing pathways had delay parameters that were about 15 ms shorter (129±20 ms) than the MG-MG (143±19) stabilizing pathway (P<0.001). The MG-TA destabilizing pathway was >40 ms longer than both stabilizing pathways (183±21, P<0.001). This additional 40+ ms loop time for the MG-TA destabilizing pathway is sufficiently long for basal ganglia involvement, compared to the shorter loop time of the MG-MG stabilizing pathway that is hypothesized to arise from reticulospinal circuits."

To make the quantitative analysis of muscle activity clearer, we have now added a callout to Figure 5 in the Results text.

> **Is the antagonist TA response truly pathological or is it perhaps compensatory?**

We consider it beyond the scope of the current paper to answer whether the antagonist TA response is pathological or compensatory. However, we believe that the delayed TA activity is a functional manifestation of the shortening response, a characteristic symptom of people with Parkinson's disease (Andrews, Neilson, and Lance, "Comparison of stretch reflexes and shortening reactions in activated normal subjects with those in Parkinson's disease." *Journal of Neurology, Neurosurgery and Psychiatry* 1973). Our preliminary data suggest that these symptoms are pathological, however to truly answer the question, one would need to follow *de novo* PD patients, and/or simulate the effects of the TA activity presuming knowledge of the balance control deficit that we are presenting for the first time here.

> **4) The authors should provide some rationale for testing in the OFF state. Given that one of the primary results is the relationship of ka' to falls (which likely occur mostly in the ON state, given that most persons with PD perform most daily activities while ON), there is a need to understand whether this abnormal TA activity also persists in the ON state. In my view, this is likely to occur because dopaminergic medications typically have a minimal effect on gait and balance, but this issue does require attention.**

We thank the reviewer for this comment. We acknowledge the limitation that the extent to which the postural abnormalities shown here would be replicated in the ON medication state is unknown – therefore the extent to which they would present in daily activities occurring over the medication cycle is unknown.

Whether testing in the OFF or ON state has more validity to fall risk is not firmly established. For example, Bloem and colleagues (*Journal of Neurology* 2001) have reported that ≈70% of parkinsonian falls occur while ON medications, but multiple reports (Foreman et al., *Parkinsonism & Related Disorders* 2011; Hoskovcová et al., *PLoS ONE* 2015) have shown that OFF state testing (specifically using gait tests) has better power to identify fallers. Further complicating matters, the extent to which different aspects of balance and gait respond to levodopa is complex (Curtze et al., *Movement Disorders* 2015) and responsiveness of some features like FOG may vary at the individual patient level (McKay et al., *npj Parkinson's Disease* 2019).

To acknowledge this limitation, we have added the following text to the limitations section:

[A]ll testing was performed in the OFF medication state, which may limit generalizability of these results to balance challenges and falls that occur during the multiple ON periods of the medication cycle experienced by most people with PD. Although it has been reported that up to 70% of falls occur while ON medications,[3] other studies have shown that OFF state measurements have more validity to falls.[67,68] Complicating matters further, medications can improve or impair different gait and balance features,[69] potentially in a patient-specific manner.[49] Testing in both the OFF and ON states could provide more insight into the extent to which the postural abnormalities shown here vary over daily periods of higher and lower fall risk.

> **5) It is not clear why only backward perturbations are included in the main analyses of the manuscript. The authors report forward perturbation data in the supplementary material. In these forward perturbation data, it does not appear that similar changes in muscle activity were observed between persons with PD and non-PD older adults. This could indicate a motor control problem specific to the TA as an antagonist (or coupling between the MG balance-corrective response and TA antagonist response). However, if this is true, it would complicate the explanation that ka' is related to falls more generally (as reported) since the reported falls likely occurred in many different directions (i.e., not only due to mechanisms similar to the backward perturbation included here). This highlights a potential issue with the generalization of the findings.**

We thank the reviewer for the comment. We agree that the asymmetry between forward and backward perturbations could indicate a motor control problem specific to TA; however, we are unable to state conclusively that this is or is not the case based on the current data. While prior research has demonstrated different neural control mechanisms for TA and MG during reactive balance (Nonnekes et al., 2013), our data does not demonstrate a clear distinction between TA and MG antagonist activity because MG antagonist activity was highly variable across participants. We used the same magnitude for forward and backward perturbations, but as forward perturbations are more difficult the muscle activity may not be directly comparable. We found some instances of abnormal MG antagonist activation in some PD participants, but not others, which are now included in the supplement. Because we did not have a full sample, we did not perform a comprehensive analysis of MG antagonist activity. To fully answer this question, we may need to modify our perturbation protocol to more consistently elicit MG antagonist activity; perturbation may have been too challenging, and caused toe-lift off which may have modified MG activity.

We have added the following text to the results:

Although we have previously reported excessive MG antagonist activity during balance tasks using a similar paradigm in PD patients in the ON medication state,37 we did not find that MG antagonist activity was elevated consistently across patients in this sample. Therefore, we did not attempt to analyze the timecourse of MG antagonist activity. Possible explanations for this finding and examples of patients with and without elevated MG antagonist activity are presented in Supplementary Materials S1.

We have now acknowledged the concerns regarding the generalizability of the deficit shown to falls circumstances other than forward perturbations with the following text added to the limitations:

…although we showed associations with overall fall frequency, the extent to which deficits in the standing balance testing paradigm used here is valid to predict falls that occur during specific circumstances, such as while standing vs. other tasks such as those including backward perturbations or gait is unknown.

> **6) As currently reported, the model is purely descriptive. A better understanding of the sensitivities of the model's parameters and the resulting implications for balance control could be achieved by perturbing the model in some way (e.g., testing different balance perturbation accelerations/magnitudes that could change the COM kinematic responses, especially in persons with PD) and testing potential predictions.**

We agree that a more complex behavioral testing paradigm could provide additional insight into mechanisms of balance deficits and falls in PD, and will be the focus of further studies. The purpose of this paper was to provide an initial cross-sectional study to demonstrate that the muscle activity in PD does in fact adhere to the CoM feedback framework necessary to justify studies as proposed by the review. If this had not been the case, the proposed studies would not

be warranted. Therefore, our work provides a fundamental first step in a line of research investigating many further studies on the sensorimotor control of balance in PD.

> **<u>Reviewer #1</u> (Minor Comments):**
>
> **Page 9, second paragraph: should "Figure 3B" be "Figure 2B"?**

We thank the reviewer for this comment. This callout was incorrectly placed. We have revised this and checked all remaining callouts in the document.

> **<u>Reviewer #2</u> (General assessment and major comments (Required)):**
>
> **Summary: This article confirms the well-known empirical result of higher co-activation of lower limb muscles in those with Parkinson's disease (Dimitrova et. al 2003). They found TA activation to be higher in the PD group than older adults, thus contributing to a balance correction inappropriately. They modified a sensory response model (SRM) to reproduce the balance correcting muscle activity in those with PD. They also correlated parameters in the model to fall occurrence. Generally, this is an interesting paper that could be improved with further discussion of the SRM and its implications**
> **Positives:**
> **1. Reproduced earlier findings of enhanced TA activation in those with PD.**
> **2. Compared muscle responses to the history of previous falls.**
> **3. Compared CoM feedback to a number of clinical assessments.**
> **4. Found that acceleration feedback, ka', is most associated with disease severity and previous number of falls.**

We thank the reviewer for their time and attention.

> **Major Concerns:**
> **1. The focus of the SRM is that PD leads to disinhibition of motor pathways that are inhibited in healthy adults. However, there is an alternative possibility... degraded estimation due to the well-established proprioceptive deficit in PD. The problem of reduced motor responses with PD is not a function of reduced muscle activation. As the authors point out with their "nuanced view", co-activation reduces the effect of appropriate responses to a perturbation. However, disinhibition of the cross-linked pathway leading to inappropriate activation of the TA may be a consequence of the fundamental problem, not the problem itself. For example, one of the most common therapies for those with PD is "Big Therapy", in which a therapist speaks forcefully to a patient in order to increase movement... and it works. This illustrates that those with PD can produce appropriate movements, given enough sensory input. Thus, the problem is the perception of their own movement, which they feel is "normal" even though it is far reduced, reflecting their proprioceptive deficit. Even though the SRM shows a nice result by accounting for the co-activation, it would be informative for the authors to discuss the estimation side of the problem and limitations of the SRM in this regard. There is nothing in the model that reflects poor estimation. It deals only with feedback about CoM position. Could a model with degraded estimation lead to such co-contraction as well? I do not expect the authors to produce or refute such a model, but to recognize in the discussion that a limitation of the**

> **SRM is that it does not incorporate a well-established deficit in those with PD and that this is another possible avenue that modeling could explore.**

We agree that the well-known proprioceptive deficit among PD may play a substantial role in balance impairments in PD. In fact, we recently showed (Bong, McKay, Factor, Ting, *Gait & Posture* 2020) that parkinsonian individuals exhibit a characteristic impairment in their ability to discriminate the direction of body motion induced by this type of perturbation. We found that parkinsonian individuals were unable to discriminate differences in perturbation direction of less than about 15°. Here, our perturbation directions were spaced at 30°, well above the perceptual threshold.

Moreover, our results indicate that the parkinsonian nervous system has adequate access to sensory signals, particularly CoM acceleration required to generate the initial bursts observed, and quantified by ka and ka'. In contrast, in our studies of proprioceptive deficit, the initial burst is absent and ka=0 (Lockhart and Ting, *Nature Neuroscience* 2007).

> **2. The SRM reproduces muscle activity in a very short time interval. Following on the point above, it may be useful to discuss how the model could be incorporated into a model that produces actual movement of the limbs.**

We agree that a generative model capable of predicting more sophisticated balance responses, including movement of the limbs, could provide additional insights into balance control. To address this concern, we have added the following text:

More complex musculoskeletal models may be required to comprehensively evaluate the impact of impaired sensorimotor feedback on fall risk in PD

> **3. The sensorimotor response model (SRM) is a post-hoc model rather than a predictive model. Does the model make predictions to test hypotheses beyond what was accounted for in the current paper?**

The main predictions that can be drawn from these results are that 1) individuals with higher k_a' measurements will be less able to respond to perturbations stronger than those used here without reactive steps or falls, and 2) individuals with higher k_a' are at increased risk of future falls. Both of these are being investigated in ongoing studies. Therefore, we consider them out of scope of the current work.

> **4. The results about the correlation between model parameters and clinical measures is mentioned only briefly in the Discussion and then discussed thoroughly in the supplementary materials. Considering this result is mentioned in the abstract, it seems to demand more space in the primary text.**

We thank the reviewer for this comment. We have expanded the discussion of clinical measures in this section and have edited the subheading to align more closely with the abstract:

Antagonist CoM acceleration feedback is most strongly associated with disease severity and reactive balance on Mini-BESTest

> **5. Balance perturbations occured in quiet stance with eyes closed while fall history was recorded as any "event that results in a person coming to rest unintentionally on the ground or another lower level", which could include during walking. Considering that many fall events could occur during walking and it is known that balance mechanisms during standing and walking are different, could this be a reason why most correlations were not significant?**

We would like to clarify that participants had their eyes open during testing. We have now specified "***with eyes open***" in the text.

Further, the reviewer is quite correct to question the extent to which feet-in-place balance testing would or should be able to predict falls that occur, say, in low light environments or due to slip hazards (like wet floors, etc.) while walking. We hypothesize that the deficit shown here would be most valid to predict falls caused by abnormal self-motion, and are investigating this in ongoing prospective studies. To acknowledge this concern, we have added the following text to the limitations section:

…although we showed associations with overall fall frequency, the extent to which deficits in the standing balance testing paradigm used here is valid to predict falls that occur during specific circumstances, such as while standing vs. other tasks such as those including backward perturbations or gait is unknown.

More generally, we acknowledge that the extent to which abnormalities revealed by feet-in-place balance testing would or should be correlated with clinical scores that predominantly measure parkinsonian signs like tremor is unknown. We have added the following text to the results to note this:

Given the sample size and limited emphasis on reactive postural control in the MDS-UPDRS-III – and to a lesser extent, Mini-BESTest, both of which are considered inadequate for assessing fall risk[42] – we did not expect to identify strong correlations with clinical variables.

> **Reviewer #2 (Minor Comments):**
>
> **1. Results Page 6 - No table referenced, "Error! Reference source not found"**

This has been corrected.

> **2. Bottom of p 8, remove "in". . "...which has been seen in younger adults in when exposed to..."**

We have revised this section.

> **3. Study group caption in Fig S3 - symbols are very small, hard to discern.**

We have increased the size of these symbols as requested.

> **Reviewer #3 (General assessment and major comments (Required)):**
>
> **McKay et al explore the hypothesis that "sensorimotor balance transformations" may be affected by PD, leading to an increase in falls. In their manuscript, the authors provide a detailed analysis of muscle activations of the TA and MG to backwards perturbations, using a compelling modeling approach. Abnormalities of antagonist muscle activation were identified as a likely contributor to falls. Further, these abnormalities in antagonist activation showed an association with disease severity.**

We thank the reviewer for their time and attention.

> **Reviewer #3 (Minor Comments):**
>
> **Introduction: "...people with PD fall most frequently during activities that require them to actively control the CoM3,4 and during which there is no change on the base of support. Examples include turning around while indoors5 or backward retropulsion while rising from a chair.6 " Some of the work cited is rather general and not specific to PD. Consider including more PD related work such as: Weaver, T. B., Robinovitch, S. N., Laing, A. C., & Yang, Y. (2016). Falls and Parkinson's disease: evidence from video recordings of actual fall events. Journal of the American Geriatrics Society, 64(1), 96-101.**

We thank the reviewer for this interesting reference. We have referenced it in the following new introduction text:

In a recent study of actual fall events captured in video recordings of individuals in long-term assistive care, people with PD were significantly more likely to experience falls provoked by incorrect weight shifting.[7]

> **Results: Please add a reference to figure 5 in the Results section.**

Thank you. We have added a callout as suggested.

> **Methods: The description of steps of data processing of kinetics, kinematics, and EMG is unspecific. Please provide additional details or reference the methods applied.**

We thank the reviewer for the comment. We have provided references and four paragraphs of additional technical details in Supplementary Materials S1 under the subheading ***"Recording and processing of kinetic, kinematic, and EMG data."*** We have also added a callout to this material in the main text.

> **The HYA are not mentioned in the Material and Methods: Recruitment section.**

We thank the reviewer for this comment. We have added the following text to this section and made some other small edits in order to incorporate it:

Younger Non-PD participants were recruited from flyers placed on college campuses. Exclusion criteria for all healthy participants were: neurological condition or significant musculoskeletal impairment as determined by the authors.

> **Falls: How do you know that all falls in the PD and non PD were "geriatric"? What about falls due to impulsivity, are these considered "geriatric"?**

We thank the reviewer for this comment. Young individuals were coded as free of falls for analysis purposes. We have removed the term "geriatric" from this section.

Abnormal center of mass control during balance: a new biomarker of falls in people with Parkinson's disease

J. Lucas McKay,[1,2,3,*] Kimberly C. Lang,[4] Sistania M. Bong,[3] Madeleine. E. Hackney,[5,6] Stewart A. Factor,[2] Lena H. Ting[1,6]

[1]Department of Biomedical Informatics, Emory University School of Medicine

[2]Jean & Paul Amos PD & Movement Disorders Program, Department of Neurology, Emory University School of Medicine

[3]Wallace H. Coulter Department of Biomedical Engineering, Emory University and Georgia Tech

[4]Graduate Division of Biological and Biomedical Sciences, Emory University

[5]Department of Medicine, Division of General Medicine and Geriatrics, Emory University School of Medicine

[6]Rehabilitation R&D Center, Atlanta VA Medical Center

*Corresponding author

Phone: +1 (404) 550-5157

E-mail: lucas@dbmi.emory.edu

Abstract

Although Parkinson disease (PD) causes profound balance impairments, we know very little about how PD impacts the sensorimotor networks we rely on for automatically maintaining balance control. In young healthy people and animals, muscles are activated in a precise temporal and spatial organization when the center of body mass (CoM) is unexpectedly moved that is largely automatic and determined by feedback of CoM motion. Here, we show that PD alters the sensitivity of the sensorimotor feedback transformation. Importantly, sensorimotor feedback transformations for balance in PD remain temporally precise, but become spatially diffuse by recruiting additional muscle activity in antagonist muscles during balance responses. The abnormal antagonist muscle activity remains precisely time-locked to sensorimotor feedback signals encoding undesirable motion of the body in space. Further, among people with PD, the sensitivity of abnormal antagonist muscle activity to CoM motion varies directly with the number of recent falls. Our work shows that in people with PD, sensorimotor feedback transformations for balance are intact but disinhibited in antagonist muscles, likely contributing to balance deficits and falls.

Introduction

Parkinson's disease (PD) causes profound balance impairments and falls, but we still know surprisingly little about how PD affects the ways in which motor outputs during balance control are organized in the nervous system based on incoming sensory information. When people maintain upright standing balance, incoming sensory information about body motion is processed by the nervous system to generate motor commands sent to activate muscles throughout the body.[1] We use the term "sensorimotor transformation" to describe this ongoing process, where sensory signals arising during a loss of balance are interpreted and used to shape the resulting balance correcting motor signals.[2] Here, our goal was to investigate whether and how sensorimotor transformations for reactive balance responses, i.e. the sensitivity of evoked muscle activity to sensory signals encoding balance error, are altered in PD.

One clue that sensorimotor balance transformations might be affected in PD is that – in addition to the fact that PD significantly increases fall risk[3] – falls in people with PD tend to occur in specific conditions involving the control of the center of body mass (CoM) that are distinct from those of falls in the general aging population. Maintaining the CoM over the base of support is critical for balance control, and dysregulation of either the CoM or the base of support can cause a fall.[4,5] However, while older adults fall most frequently due to slips and trips that cause a sudden change in the area of the base of support, people with PD fall most frequently during activities that require them to actively control the CoM[3,4] and during which there is no change on the base of support. Examples include turning around while indoors[5] or backward retropulsion while rising from a chair.[6] In a recent study of actual fall events captured in video recordings of individuals in long-term assistive care, people with PD were significantly more likely to experience falls provoked by incorrect weight shifting.[7] Such activities require that the nervous system sense and monitor the body and generate appropriate motor responses, suggesting that there might be impairments in sensorimotor balance transformations that are more pronounced in PD than in the general geriatric community.

Laboratory studies have also shown that motor signals to muscles are abnormal in people with PD. When muscles are stretched and lengthened, spinal reflexes typically increase

the activity in the muscle, helping to maintain limb posture. (These muscles are often referred to as "agonists.") Work in the early- to mid-1900s described paradoxical "shortening reactions" in PD, in which muscles activate when they are shortened during passive movements, counteracting the stretch reflex response and reinforcing the new imposed position.[8] Later studies showed that PD patients exhibit a complex pattern of motor signals during voluntary reaching, in which agonist and antagonist muscles activate in series of multiple bursts rather than the simple three-burst pattern seen in people without PD.[9] Studies using moving platforms to perturb standing balance show the main responses for balance corrections during support-surface translations are in agonist muscles that are first stretched by the balance perturbations. However, in PD, the antagonist muscles that are shortened by perturbations in PD patients are also activated, counteracting the corrective torques generated by the agonist muscles lengthened by balance perturbations.[10–14]

In animals and healthy young human subjects, motor signals to agonist or "prime mover" muscles are generated based on sensory feedback of CoM motion during balance. However, it is unclear whether this sensorimotor transformation is preserved in PD. When standing balance is disturbed, motor signals to agonist muscles are created with a sensorimotor feedback transformation in which agonist muscles are activated in proportion to CoM motion.[2,15,16] Patterns of magnitude and timing of muscle activation can be explained with a small set of feedback gains that describe sensitivity to CoM acceleration, velocity and displacement (k_a, k_v, and k_d); and a delay (λ) to account for sensorimotor processing and transmission time.[17–19] We know from animal studies that this activity depends on brainstem and spinal networks, with important roles for subcortical structures including thalamus and subthalamic nucleus (STN).[20,21] What remains unclear is whether the sensorimotor transformation for balance control is disrupted in PD, but clearly these subcortical structures, i.e. the thalamus and STN, are affected.[22] Further, even if CoM feedback is abnormal in PD the relevance to functional outcomes like falls is unknown. For example, PD is associated with additional coactivation during walking, but the presence of coactivation is weakly related to gait quality.[23]

The sensorimotor processing underlying motor signals to antagonist muscles during balance are not understood, but may arise from similar CoM sensorimotor feedback signals driving agonist muscles. An antagonist activation pathway based on CoM feedback analogous to that of agonist muscles seems plausible, as antagonist muscles are frequently activated during motor tasks in uncertain environments.[24] If so, paradoxical antagonist activity in PD may be generated by otherwise healthy sensorimotor processes, for example, processes that have been released from tonic inhibition[25] or activated by descending signals from higher motor centers with abnormal timing,[26] given that basal ganglia dysfunction can produce both hypo– and hyper–kinetic signs.[27] In one previous study, a CoM feedback scheme was used to explain changes in components of antagonist muscle responses over the course of motor adaptation;[16] however, whether antagonist activity during balance in general or in the parkinsonian state can be described by CoM feedback is unknown.

Here, we tested how sensorimotor transformations both driving agonist and antagonist muscles during balance control are disrupted in PD, and related these changes to falls as well as clinical variables reflecting different aspects of the underlying pathophysiology. Using support-surface perturbations to standing balance, we disturbed the position of the CoM over the base of support. We tested whether sensorimotor feedback transformation identified previously in younger adults could explain the generation of both agonist and antagonist muscle activity in people with PD and age-similar older adults without PD (Non-PD), and whether there were abnormalities specific to PD. We further tested whether the presence and severity of abnormal CoM feedback was associated with the presence and number of previous falls over the prior 6 months. Finally, we tested whether the abnormal sensorimotor transformation features were associated with clinical variables reflecting PD severity and duration, and with clinical measures of balance impairment.

Overall, we found that sensorimotor transformation from CoM sensory information to motor signals to *tibialis anterior* was elevated in PD vs. Non-PD, with increased amounts of feedback and a longer processing time (by ≈20 ms) than the sensorimotor transformation from CoM to agonist muscles. Further, this abnormal sensitivity to CoM acceleration was associated with a history of frequent falls. Taken together, abnormal CoM feedback likely contributes to

balance impairments and may arise from abnormal activity of supraspinal centers that interact with brainstem and spinal networks previously hypothesized to mediate sensorimotor transformations for balance in young healthy individuals and animals.

Results

Participants and setting

We examined temporal patterns of muscle activation evoked by support surface translation perturbations in N=44 people with mild-moderate Parkinson disease (PD, average disease duration, 7.4 ± 4.8 y, range 8 m-21 y) and N=18 matched neurotypical individuals (Non-PD, Table 1). Most PD patients (62%) were of the Postural Instability and Gait Disability (PIGD) PD symptom phenotype,[28] and most (66%) did not report freezing of gait (FOG). With the exception of 2 PD patients early in the disease course who had not yet started pharmacotherapy, the remainder were prescribed antiparkinsonian medications (average levodopa equivalent daily dose (LED)[29] 726 ± 357 mg, range 280-1800 mg). All assessments were performed in the practically-defined 12-hour OFF medication state.[10] None had previously undergone functional neurosurgery. Detailed medication information is provided in Supplementary Materials S1.

No statistically-significant differences were observed between PD and Non-PD participants on age, sex, height, weight, or overall cognition (Montreal Cognitive Assessment, MoCA[30]). Participants were cognitively normal according to established criteria.[31] However, PD participants exhibited poorer overall performance on clinical balance and gait outcomes, with statistically-significant impairment on the current clinical standard Mini-BESTest[32] (2.6 points, P=0.017, independent samples *t*-test, unpooled variance assumption) as well the Fullerton Advanced Balance Scale (FAB,[32] 3.4 points, P=0.028), the Dynamic Gait Index (DGI,[33] 1.9 points, P=0.043), and the Berg Balance Scale (BBS,[34] 2.1 points, p=0.040). Some analyses also considered a reference sample of N=6 young healthy individuals recruited from a college campus (HYA, 4 female, 21 ± 2 y). No statistically-significant differences were identified between groups on sex, height, MoCA score; however, younger participants weighed less (61 ± 12 vs. 77 ± 14 kg; P=0.039).

In PD, balance-correcting muscle activity is normal, but antagonist TA muscle activity is elevated

We assessed each participant with support surface translation perturbations delivered in unpredictable order in directions evenly spaced throughout the horizontal plane (Figure 1A). The apparatus and testing paradigm have been described previously.[10] In order to assess the balance control system without inducing actual falls, the perturbation parameters were refined over a series of studies in Parkinson's patients in both the OFF and ON medication states to be very near, but to not exceed, the level at which they could maintain balance without stepping.[10,35–37] We analyzed backward platform translation trials (240°, 270°, 300°) that initially lengthened the plantarflexor *medial gastrocnemius* (MG) (Figure 1B, *start* epoch). This is considered a balance-correcting response; MG activity was normalized to the maximal response observed in these trials. We often observed a coactivation response in the dorsiflexor *tibialis anterior* (TA), which is considered to be an antagonist until the end of the perturbation when the platform decelerates (Figure 1B, *stop* epoch). TA muscle activity was normalized to the maximal activity observed in forward perturbations (60°, 90°, 120°) where it acts as the agonist for the primary balance-correcting response.[10]

Balance-correcting MG responses to backward translation perturbations in older adults with and without PD were very similar to that observed previously in young healthy individuals[10,15,16] and in cats,[2] activating when the MG muscle is stretched. MG exhibited an initial burst at latency ≈100-150 ms following the onset of platform motion and initial acceleration of the CoM followed by a plateau of activity of ≈200-400 ms duration comparable to the duration of time in which the CoM was being displaced with positive velocity forward with respect to the ankles (Figure 1B, left).

Individuals with PD also often exhibited a large initial burst of antagonist TA muscle activity during backward perturbations that oppose the balance-correcting actions of the MG muscle (≈100-150 ms) when it was shortened by perturbations (Figure 1B, right). In neurotypical older participants, the TA response to posterior perturbations was typically quite similar to that previously observed in young healthy individuals, and was primarily characterized by a response to the *deceleration* of the platform ≈600 ms after perturbation

onset, with little if any activity above background levels during earlier phases. However, in PD patients, the TA response was often characterized by a strong initial burst at ≈100-150 ms latency very comparable to that observed in MG, so that in some cases TA appeared to activate in a pattern of magnitude and timing almost identical to that of MG.

Abnormal antagonist TA activity during backward perturbations was significantly elevated in PD compared to matched neurotypical individuals (56%, P<0.05, ANOVA, post hoc tests; Figure 1C) as well as to young healthy individuals (P<0.01). Difference in peak muscle activity 100-600 ms after perturbation onset were only found in antagonist TA activity, and not balance-correcting MG activity.

Although we have previously reported excessive MG antagonist activity during balance tasks using a similar paradigm in PD patients in the ON medication state,[37] we did not find that MG antagonist activity was elevated consistently across patients in this sample. Therefore, we did not attempt to analyze the timecourse of MG antagonist activity. Possible explanations for this finding and examples of patients with and without elevated MG antagonist activity are presented in Supplementary Materials S1.

Hypothesized sensorimotor feedback pathways decomposed balance correcting and antagonist muscle activity in healthy aging and in PD

We hypothesized that balance correcting and antagonist muscle activity across aging and disease could be explained by a common underlying sensorimotor transformation between CoM motion and muscle activation. To explicitly test our hypothesis, we reconstructed the entire timecourse of balance correcting and antagonist muscle activity using a model that was previously used to reproduce balance-correcting muscle activity in healthy and impaired animals as well and in healthy young humans[2,17] (Figure 2).

In the sensorimotor response model (SRM), balance-correcting muscle activity (Figure 2AB, green) is reconstructed by a weighted sum of horizontal plane CoM acceleration (a), velocity (v), and displacement (d) occurring ≈100 ms earlier, which acts to stretch the muscles. Thus, three feedback gain parameters (or weights, k_a, k_v, k_d) and a lumped time delay (λ) for each muscle are identified by minimizing the error between recorded and reconstructed EMG

signals. The resulting parameters quantify the contributions of acceleration, velocity, and displacement sensory signals to balance correcting responses.

It was evident that the established SRM would be unable to explain antagonist TA activity that occurs while it is shortening, rather than stretching (cf.[16]). To explicitly account for abnormal antagonist TA activity in PD during backward perturbations, we extended the SRM with a new antagonist pathway in which sensory signals driving balance-correcting MG activity also activate TA (Figure 2AB, red). TA activation during shortening has been previously reported in our laboratory when younger adults were exposed to novel or unpredictable perturbations.[16] Here, we extended the SRM model to also include parameters associated with the activation of TA as an antagonist ($k_a{}'$, $k_v{}'$, $k_d{}'$, λ'). These parameters explicitly dissociate hypothesized sensory signals underlying the initial, antagonist TA muscle activity from later, balance-correcting TA muscle activity when the support-surface decelerates at the end of the perturbation.

The addition of the antagonist pathway significantly improved the ability of the SRM to explain PD antagonist TA activity, improving adjusted coefficient of determination (R_a^2) by 0.18±0.15 on average (P<0.001) and increasing peak antagonist activity 100-600 ms after perturbation onset by 0.23±0.11 normalized units (nu) on average (P<0.001). When we performed similar comparisons on PD MG antagonist activity during forward perturbations, we noted similar improvements in fits (improvement in R_a^2, 0.13±0.14, P<0.001; increase in early antagonist activity, 0.14±0.10 nu, P<0.001); however, antagonist activity remained significantly higher in TA (64%, P<0.001) than in MG (Supplementary Materials S1).

The extended SRM accounted for both balance-correcting and antagonist muscle activity in during backward perturbations with long-latency delayed feedback of CoM kinematics, with grand mean VAF of 82±6% and 81±7% in TA and MG, respectively. These values were generally comparable (attenuated by ≈6% and ≈1%, respectively) to those in an earlier study of young healthy participants[1] and were considered acceptable. The only statistically-significant differences in VAF identified across groups was a small decrease (1%) was among young compared to older participants in TA (Supplementary Materials S1).

TA antagonist CoM acceleration feedback is increased in PD and in aging

We hypothesized that the large initial burst of antagonist TA muscle activity during backward perturbations reflected increased sensitivity to peak CoM acceleration. We observed no meaningful differences in peak CoM acceleration (-3%, P=0.18, ANOVA), velocity (+7%, P=0.07), or displacement (-3%, P=0.73) across groups (Supplementary Materials S1). Therefore, the initial burst could only be reconstructed by increasing the sensitivity of the muscle activity to the initial acceleration.

Consistent with our hypothesis, ANOVA and post-hoc tests showed that TA antagonist CoM acceleration feedback parameter $k_a{}'$ was significantly higher among PD compared to Non-PD (increased by 95%, P<0.001) as well as among older participants (PD and Non-PD) compared to the young group (+239%, P<0.001) (Figure 2C). Among the PD group, there was no statistically-significant variation in $k_a{}'$ across PD phenotypes (P=0.94, ANOVA on PIGD vs. TD vs. Indeterminate; See Supplementary Materials S1).

Taken together with imaging studies in older adults with and without PD, the elevated values of $k_a{}'$ in PD suggest that – in addition to the dopaminergic degeneration characteristic of PD – patients with high values of $k_a{}'$ likely also had substantial cholinergic deficits.

Other TA CoM feedback parameters are affected in aging

Several other SRM gain parameters varied strongly across age groups, with significantly higher values of antagonist displacement gain $k_d{}'$ (+854%, P<0.001), as well as of balance correcting acceleration gain k_a (+334%, P<0.001) and of balance correcting velocity gain k_v (+100%, P=0.003) among the older participants compared to the young group. Compared to the young group, among the older participants values of antagonist delay λ' were significantly longer (183 vs. 136 ms, P=0.018) but values of balance correcting delay λ were significantly shorter (129 vs. 151 ms, 14% P=0.014). No statistically-significant differences among groups were observed in SRM parameters describing balance correcting activity in TA during perturbations that caused it to lengthen, or in MG activity. (See Supplementary Materials S1.)

TA antagonist CoM acceleration feedback is increased with fall history and with number of previous falls

Multivariate analyses showed that SRM parameter k_a' was strongly associated with the presence and number of previous falls. Previous falls were significantly more prevalent among the PD group than among other participants (47% vs. 12%; P=0.008; compare blue to brown bars, Figure 3A). Compared to participants with no fall history, k_a' was significantly increased (115%, P<0.001, ANOVA; Figure 3B1) among those with ⩾2 falls in the prior 6 months after controlling for age, sex, presence of PD, and presence of FOG. k_a' was also significantly increased among participants with PD (37%, P=0.042; Figure 3B2) and tended to increase with age (≈8%/decade, P=0.116) in multivariate analysis. No significant effects of sex (P=0.742) or presence of FOG (P=0.584) were identified. No significant differences were identified in k_a' between participants with 1 and 0 fall over the prior 6 months (P=0.721).

Although a maximum of only 1 fall over the prior 6 months was reported in the Non-PD group, average fall frequency was substantially higher in the PD group. The average 6 month fall frequency was 12, and the maximum was 180, corresponding roughly to biweekly and daily falls. The long tail of the fall frequency distribution provided strong support for the use of a negative binomial model to represent the data.[38] Negative binomial regression is common in epidemiological studies and has been frequently used for fall frequency data.[39–41] Multivariate negative binomial regression identified a strong nonlinear relationship between fall frequency and k_a', even after controlling for known fall risk factors (Figure 3C). The identified regression coefficient between fall frequency and k_a' was $\beta_{ka'} = 0.66$ (95% CI 0.32–1.00; P=0.0002). The model indicated that although among young participants an increase in k_a' from the 15th to 85th percentile had no effect on the expected number of falls (0), among the older Non-PD group, a similar increase in k_a' was associated with an increase in the expected number of falls from 0 to 3, and among PD, a similar increase in k_a' was associated with an increase in the expected number of falls from 1 to 11. Estimated fall frequencies as functions of k_a' for different estimated participants are shown in Figure 3C.

TA antagonist CoM acceleration feedback is most strongly associated with disease severity and reactive balance on Mini-BESTest

After controlling for effects of age, k_a' was most strongly associated with clinical indicators of disease severity and with a behavioral measure of reactive postural control on the Mini-BESTest (Figure 4). Given the sample size and limited emphasis on reactive postural control in the MDS-UPDRS-III – and to a lesser extent, Mini-BESTest, both of which are considered inadequate for assessing fall risk[42] – we did not expect to identify strong correlations with clinical variables. In particular, ≈40% of MDS-UPDRS-III items measure tremor severity or upper limb motor performance, which are not commonly considered to be associated with falls.

We considered Pearson product-moment correlation coefficients ⩾0.10 to be non-negligible, according to criteria proposed by Cohen.[2] Univariate models (Figure 4A) identified non-negligible correlations between k_a' and clinical measures of disease severity and reactive balance, including: increased age (r = 0.18); increased disease severity as indicated by increased disease duration (r = 0.26), amount of dopaminergic medication (LED; r=0.19), total symptoms (MDS-UPDRS-III total score; r = 0.19); and more impaired postural control on MDS-UPDRS-III (r = 0.10) and Mini-BESTest (r = -0.16).

It was notable that no association was identified between k_a' and overall balance ability as indicated by total Mini-BESTest score (r = -0.02), potentially because most patients were quite high-performing on this test, with >75% above clinical cutoff values for fall risk.[44] More severe k_a' was also associated with more impaired cognition on the MoCA (r = -0.12), which was notable given that >95% of the sample MoCA scores were ⩾26, indicative of normal cognition in PD.[3] After adjusting for effects of age, the only associations that remained non-negligible were with PD duration, LED, and Mini-BESTest reactive postural control (Figure 4B, crosses). No identified correlations were statistically significant.

TA antagonist CoM acceleration feedback delay times are consistent with long-loop activity

The delay between CoM kinematics and muscle activity in the destabilizing pathway was substantially longer than in the stabilizing pathways, suggesting the involvement of higher-level neural influences (Figure 5). Identified SRM delay parameters in each of the identified pathways were not different in PD versus non-PD groups (p=0.54). The TA-TA stabilizing pathways had delay parameters that were about 15 ms shorter (129±20 ms) than the MG-MG (143±19) stabilizing pathway (P<0.001). The MG-TA destabilizing pathway was >40 ms longer than both stabilizing pathways (183±21, P<0.001). This additional 40+ ms loop time for the MG-TA destabilizing pathway is sufficiently long for basal ganglia involvement, compared to the shorter loop time of the MG-MG stabilizing pathway that is hypothesized to arise from reticulospinal circuits.

Discussion

Here we demonstrate that in PD, sensorimotor transformations during perturbations to standing balance are intact but dysregulated, generating temporally precise but spatially diffuse muscle activity in response to CoM motion. We show that in older adults with and without PD, the sensorimotor control of agonist muscles exhibits characteristic changes compared to that in young healthy individuals. Further, in older adults with PD, antagonist muscles also exhibit large transient responses when shortened that are time-locked to CoM motion. This abnormal activity presumably hinders balance corrections by stiffening the joints and reducing postural robustness. The presence and magnitude of abnormal antagonist activity is associated with the number of previous falls, which would be the case if this activity were a cause of falls. Among the clinical variables examined, the strongest association was observed between abnormal antagonist activity and disease duration.

This suggests that CoM sensorimotor control in PD may continue to degenerate after the initial degeneration of dopaminergic neurons in the basal ganglia is largely complete. At least 80% of dopaminergic cells are gone by the time of diagnosis in most PD patients. However, we found an effect of PD duration among patients for whom this process was in later

stages, and for whom there were almost certainly no dopaminergic cells remaining. Therefore, we speculate that these processes continue after dopaminergic cell loss is complete.

PD does not grossly impact the sensorimotor processes used to activate prime mover muscles during balance control. Our results show that temporal patterns of agonist muscle activity in older adults with and without PD exhibit a precise relationship to sensory inflow during balance perturbations. Consistent with previous descriptions of EMG activity in PD,[11] we found that active sensorimotor feedback control of agonist muscles (TA and MG) was generally very similar in PD compared to healthy older adults. We used the same sensorimotor transformation previously demonstrated in younger adults and in animals to successfully reproduce the timing and magnitude of muscle activity based on the acceleration, velocity, and displacement of the CoM elicited during perturbations. Contrary to the idea that postural responses in PD are delayed, we found no differences in the delay between CoM motion and muscle activity in older adults with and without PD. In fact, the delays were slightly shorter than those found previously in younger adults. This shorter delay may reflect increased sensory drive due to the increased difficulty of the task for older adults.

Abnormal antagonist TA activity in PD appears to be generated by temporally precise sensorimotor signals arising from and driving prime mover muscles, but routed differently through the nervous system and with a slightly longer delay. An important distinction from agonist muscle activation – in which muscles are activated by sensory signals originating in the same muscles[2,15] – is that the antagonist TA response depended on sensorimotor feedback originating in different muscles initially lengthened by the perturbations. To explain this antagonist activity, we added additional feedback channels arising from the agonist MG to describe a polysynaptic feedback arc to the antagonist TA. The sensitivity of the antagonist TA to the acceleration of the CoM revealed the largest differences in muscle activity between older adults with and without PD. Importantly, these signals could not have originated within TA, which is slack during the initial portion of this perturbation. Instead, they likely arise from muscle spindle proprioceptive signals[2,23] within the MG and within other muscles within the limbs – and potentially torso and other areas – that are stretched due to the perturbations and which exhibit similar feedback-mediated responses.

Our results suggest that falls in PD may result from abnormal antagonist activity that interferes with otherwise appropriate responses, rather than the inability to activate prime movers. Postural reactions in PD are often described as "slower" than in individuals without PD,[45] a useful description that is consistent with well-documented delays in reaction time PD patients demonstrate in many tasks.[46–48] However, our results favor the more nuanced description that postural responses are "slow[er] to develop force" than in controls,[45] because we found that the most prominent feature of postural responses in PD was the abnormal activation of antagonist muscles that rendered the ongoing balance correcting response ineffective, rather than absent or delayed responses in agonist muscles. We found that antagonist sensitivity to CoM acceleration feedback was significantly associated with both the presence of fall history and the number of previous falls in the 6 months prior to study enrollment, even after controlling for fall risk factors (age, sex, and FOG). Increased antagonist muscle activity could hinder the generation of corrective joint torques required to stabilize the CoM during standing as well as voluntary movements.[4,5] The same mechanisms for stabilizing the CoM are likely used during, as corrective muscular responses following perturbations during locomotion are superimposed upon the ongoing locomotor pattern in both the stance and swing limb.[49] Abnormal CoM control may therefore represent a fall risk factor in addition to other factors such as freezing of gait,[50,51] rigidity,[52] and cognitive impairment.[53]

One explanation for these data could be that networks enabling the routing from agonist to antagonist muscles exist in the healthy nervous system, and are selectively disinhibited during specific task conditions in youth and healthy aging, but continually disinhibited in PD. Typically, the control of antagonistic pairs of muscles such as TA and MG is organized reciprocally, so that activation of one inhibits the other.[54] However, in healthy individuals, physiological mechanisms exist that enable co-contraction between these muscles,[55] particularly in unpredictable[16] or unstable environments,[56] and in situations of increased postural threat, such as while standing at an elevated height.[57] Possibly this pathway is disinhibited only when coactivation is necessary in the healthy nervous system, but that is disinhibited more broadly in PD. Top-down "gating" of sensory input has been identified throughout the mammalian nervous system.[58] In PD, whether the sensorimotor control of

antagonist muscles is abnormal in general is unknown. However, previous results in other muscles[10,11] have shown abnormal overall activity similar to that observed here in TA. Notably, although we did not find a robust increase in antagonist activity in MG in coarse analyses of overall muscle activity– and therefore did not perform the entire SRM model fit on this data – anecdotally, some patients exhibited strong antagonist responses in this muscle. Some antagonist activity in MG would be expected for concordance with previous results. Individual cases with and without abnormal antagonist activity in MGAS are described in Supplementary Materials S1.

Taken together with other studies, these results suggest that sensorimotor control in PD may continue to degenerate due to changes in subcortical mechanisms even after the initial degeneration of dopaminergic neurons in the basal ganglia is largely complete. There are two aspects of these results that point to a neurophysiological substrate other than the basal ganglia for these deficits. First, these patients were fairly advanced (10 y), and we found a strong effect of PD duration. At this duration, the changes in the basal ganglia are long over. Therefore, to find an effect of duration suggests that there is dependence on pathological mechanisms that continue to progress after 5-10 years. These are largely non-dopaminergic.[59,60] Second, based on other studies using similar patient cohorts, it is very likely that patients with high values of antagonist activity had considerable cholinergic deficits in regions including the thalamus.[61–63] The latency on the antagonist pathway we identified is consistent with involvement of supraspinal centers.[56] In particular, longer-latency stretch responses in TA (>95 ms) can be modulated with transcranial magnetic stimulation, which suggests involvement of supraspinal centers.[64] The involvement of the thalamus in particular is suggested by animal work, which provides some evidence that the thalamus is necessary for the appropriate generation of postural response muscle activity[20] and receives monosynaptic sensory feedback from the spinal cord.[65]

Limitations

There are several limitations to this study of note. First, the lack of imaging data prevents us from more concretely identifying the neuroanatomical substrates of these deficits.

Although the ability to image changes in the basal ganglia and other brain regions associated with Parkinson's disease remains limited (cf.[61,62,66,67]), without any such data we are forced to speculate on the neuroanatomical substrates involved. These results suggest that investigations incorporating the balance testing approaches used here in addition to neuroimaging approaches are warranted. Second, the use of retrospective measurements of fall frequency allows the possibility that previous falls could have caused the abnormalities in CoM control we report. Ongoing prospective studies will guard against this possibility. Third, all testing was performed in the OFF medication state, which may limit generalizability of these results to balance challenges and falls that occur during the multiple ON periods of the medication cycle experienced by most people with PD. Although it has been reported that up to 70% of falls occur while ON medications,[3] other studies have shown that OFF state measurements have more validity to falls.[68,69] Complicating matters further, medications can improve or impair different gait and balance features,[70] potentially in a patient-specific manner.[50] Testing in both the OFF and ON states could provide more insight into the extent to which the postural abnormalities shown here vary over daily periods of higher and lower fall risk. Additionally, although we showed associations with overall fall frequency, the extent to which deficits in the standing balance testing paradigm used here is valid to predict falls that occur during specific circumstances – such as while standing vs. other tasks such as those including backward perturbations or gait – is unknown. More complex musculoskeletal models may be required to comprehensively evaluate the impact of impaired sensorimotor feedback on fall risk in PD.

Conclusions

These results demonstrate that the sensorimotor feedback control of agonist muscles is affected by healthy aging, and that the sensorimotor feedback control of antagonist muscles is affected by PD. Abnormal sensorimotor feedback control of antagonist muscles is a potential cause of falls in PD. Abnormal sensorimotor feedback control of antagonist muscles is associated with increased progression in PD, and may involve non-dopaminergic centers. Clinical evaluations of balance in PD in neurological testing involving involuntary

perturbations of the CoM forward with respect to the ankles could reveal important features of impaired balance.

Materials and Methods

Recruitment

Participants with PD were recruited from healthcare centers and patient advocacy organizations in the Atlanta area. Healthy participants were recruited from older adult advocacy groups, referral from researchers at Emory University and Georgia Tech, and from flyers placed on college campuses. All PD patients met the following inclusion criteria: Hoehn and Yahr Stages I-IV, age ⩾ 35 years, ability to walk with or without assistive device ⩾ 10 feet, normal perception of vibration and light touch on feet. Exclusion criteria for PD patients were: significant neurological or musculoskeletal impairment as determined by the authors. Older Non-PD participants were recruited to be similar in age and sex to the PD group, but were not matched individually to each patient. Younger Non-PD participants were recruited from flyers placed on college campuses. Exclusion criteria for all healthy participants were: neurological condition or significant musculoskeletal impairment as determined by the authors. Data were collected from January 2014-July 2018. Some participants (23/44 PD, 11/18 Non-PD) were recruited as part of a rehabilitation study, other outcomes of which have been and will be reported separately.[10]

Assessment protocol

All participants were assessed with common clinical measures of balance ability, PD severity and with a brief cognitive screen.[35] PD patients were instructed to abstain from antiparkinsonian medications for at least 12 hours prior to assessment but to continue taking all other medications on their typical schedule. PD severity was assessed with the original or MDS-revised Unified Parkinson Disease Rating Scale, part III (MDS UPDRS-III), and items from part II (activities of daily living) relevant to tremor and postural and gait disability.[71] Assessments were videotaped and rated by a movement disorders specialist (author S.A.F.) or MDS certified rater (author M.E.H.). UPDRS-III scores were converted into equivalent MDS

UPDRS-III scores according to methods established in the literature.[72] Individual UPDRS-III items were used to establish Tremor-dominant (TD-PD) or Postural Instability and Gait Difficulty (PIGD-PD) PD phenotype.[28] Participants with PD were classified as "freezers" based on scores ⩾2 on the Freezing of Gait questionnaire (FOG-Q)[6] item 3, indicating freezing episodes "about once per week. N=3 patients for whom FOG-Q was unavailable were classified based on UPDRS II item 14, indicating "occasional freezing when walking." Behavioral balance outcomes included the Mini-BESTest,[32] Berg Balance Scale (BBS),[34] Fullerton Advanced Balance Scale (FAB),[32] and Dynamic Gait Index (DGI).[33] Global cognitive status was assessed with the Montreal Cognitive Assessment (MoCA).[74] Non-PD and PD participants were interviewed with a standardized instrument for health history including the presence of previous falls.[10] Clinical information was abstracted from medical records for 21/44 PD patients; for the remainder of patients and for all neurotypical participants this information was obtained via self-report.

Medical and other exclusions

Data were initially available for N=66 participants with PD and N=32 Non-PD participants. PD participants were excluded from analyses due to: MoCA score < 25 indicative of MCI[74] (N=10); altered diagnosis after enrollment (N=5); inability to complete assessment (N=4); equipment problems leading to invalid EMG or other laboratory data (N=3). Non-PD participants were excluded from analyses due to: MoCA score < 25 indicative of MCI[74] (N=5); neurological condition disclosed after enrollment (N=1); inability to complete assessment (N=1); equipment problems leading to invalid EMG or other laboratory data (N=7). After applying exclusions, data of N=44 PD patients and n=18 neurotypical participants were available for analysis. Complete case analyses were used in the event of missing data.

Reactive balance assessments

Reactive balance assessments were conducted with methodology used previously in earlier studies of PD patients.[10] Participants stood barefoot on two force plates installed in a translating platform with their arms crossed across their chest, feet parallel and eyes open and focused on a large landscape poster 4.6 m ahead. Participants were exposed to between 36 and 60 ramp-and-hold translations of the support surface (peak acceleration: 0.1 g; peak velocity:

25 cm/s; peak displacement: 7.5 cm; time from initial acceleration to initial deceleration 450 ms) with direction selected randomly among 12 directions evenly distributed in the horizontal plane and unpredictable by the participant. Stance width was fixed at 26 cm between the medial malleoli. Kinematic, kinetic, and EMG data were collected and synchronized as in previous studies.[10] EMG was recorded bilaterally from muscles in the legs and trunk and processed off-line (high-pass, 35 Hz; de-mean; rectify). EMG and other analog signals were sampled at either 1080 Hz or 1200 Hz depending on equipment. Body segment kinematic trajectories were collected at 120 Hz. CoM displacement and velocity in the horizontal plane were calculated from kinematic data as a weighted sum of segmental masses, and CoM acceleration in the horizontal plane was calculated from recorded horizontal-plane forces.

Data processing

Analyses were conducted on bilateral recordings from *tibialis anterior* (TA) and *medial gastrocnemius* (MG). After high-pass filtering, full-wave rectification, and low-pass filtering, EMG, kinematic, and kinetic signals were aligned to perturbation onset and averaged over replicates of each perturbation direction separately for each participant.[10,15,35,75] Additional details are provided in Supplemental Materials S1. The resulting average EMG recordings of each muscle of each participant (TA from the left and right leg as well as MG from the left and right leg) then were normalized to the maximum value observed during a wide window 80–425 ms after perturbation onset in all perturbation directions examined. This window encompassed medium- and long-latency postural reflex responses. Analyses were performed to perturbation directions within ±30° of the cardinal anterior and posterior directions, which elicit strong bilateral responses in both muscles studied.

Sensorimotor response modeling

To quantify whether abnormalities in surface electromyographic activity associated with Parkinson disease reflected central changes in the sensorimotor transformation between center of mass kinematics and recorded muscle activity, we computed relationships between measured patterns of electromyogram magnitude and timing with recorded center of mass kinematic signals using our sensorimotor response model.[2,15,17]

Sensorimotor response model parameters that best reproduced the entire time course of muscle activity were found by minimizing an error term calculated between recorded EMG and reconstructed signals. The error term was quantified as the sum of squared errors at each time sample and the maximum observed error:

$$\min_{k,\lambda}\left\{\mu_s\int_0^{t_{end}} e^2 dt + \mu_m \max(|e|) + \mu_k k^T k\right\}$$

where first term penalizes squared error e^2 between averaged and simulated muscle activity with weight μ_s, the second term penalizes the maximum error between simulated and recorded muscle activity at any point with weight μ_m, and the third term μ_k is a nuisance term that penalizes the magnitudes of gain parameters k in order to improve convergence when feedback channels do not contribute to reconstructed electromyogram signals. The ratio of weights μ_s:μ_m:μ_k was 1:1:1e-6. Additional details are provided in Supplementary Materials S1.

Balance-correcting CoM feedback

To test whether feedback rules used to active muscles in response to perturbations in the healthy nervous system were altered in PD, we compared the ability of two primary models to reproduce muscle activation patterns based on CoM motion. In both, the overall hypothesis was that CoM kinematic signals are linearly combined in a feedback manner to generate muscle activity.

In the first model, balance-correcting CoM feedback, recorded EMG responses were reconstructed using kinematic signals describing horizontal plane CoM acceleration (a), velocity (v), and displacement (d), that were each weighted by a feedback gain (k_a, k_v, k_d), summed, and subjected to a common time delay (λ) to simulate neural transmission and processing time:

$$EMG_{recon} = \lfloor k_d d(t-\lambda) + k_v v(t-\lambda) + k_a a(t-\lambda)\rfloor \qquad (1)$$

with the total summed signal subjected to a rectification nonlinearity in order to represent excitatory drive to motor pools:

$$\lfloor\cdot\rfloor = max(0,\cdot)$$

For TA, the signals (a, v, d) describe motion of the CoM backward with respect to the ankles, which cause TA to lengthen, and are hypothesized to be encoded primarily in TA muscle spindles.[76] Conversely, for MG, the signals describe motion of the CoM forward with respect to the ankles, which cause MG to lengthen, and are hypothesized to be encoded primarily in MG muscle spindles. In the model, this is implemented by multiplying kinematic signals recorded in the extrinsic coordinate system of the laboratory by an appropriate factor (1 or -1 given the default coordinate system in our laboratory) so that motion backward with respect to the ankle corresponds to positive values of a for TA and so that motion forward with respect to the ankle corresponds to positive values of a for MG. We assumed transient acceleration encoding was limited by muscle spindle cross-bridge cycling,[77,78] and implemented this by setting acceleration feedback to zero after a fixed time window[17] (see Supplementary Materials).

Nonspecific CoM feedback

Because the early antagonist TA activity in PD during perturbations when the CoM was initially displaced forward showed some striking similarities to the patterns of magnitude and timing observed in the agonist MG, we also tested a second feedback scenario in which the kinematic signals that are typically used to activate MG could explain TA magnitude and timing. In this scenario, we added additional feedback channels for signals describing motion of the CoM forward with respect to the ankles, which cause TA to shorten (as well as MG to lengthen), and are hypothesized to be encoded primarily in MG muscle spindles:

$$EMG_{recon} = \lfloor k_d d(t-\lambda) + k_v v(t-\lambda) + k_a a(t-\lambda) \rfloor + \lfloor -k_d' d(t-\lambda') - k_v' v(t-\lambda') - k_a{}' a(t-\lambda') \rfloor \quad (2)$$

In addition to the components of Equation 1, additional delayed feedback gains $(k_a{}', k_v{}', k_d{}')$ and an independent delay λ' have been added to describe these additional signals. For TA, signals (a, v, and d) describe motion of the CoM backward with respect to the ankle that lengthen TA, and signals ($-a$, $-v$, and $-d$) describe motion of the CoM forward with respect to the ankle that lengthen MG.

Statistical methodology

Statistical tests were performed in Matlab r2018b, SAS University Edition 7.2, or R 3.6.1. Tests were considered statistically-significant at P⩽0.050. Tests of different kinematic variables (e.g., peak CoM acceleration or peak CoM velocity) or of different model parameters (e.g., k_a' or λ) were assumed to evaluate independent null hypotheses and were performed without adjustment for simultaneous inference.[7] Summary statistics are presented as sample mean±sample standard deviation, sample mean (95% confidence interval), or count (percent).

Participants and setting

Comparisons of clinical and demographic variables between groups were performed with independent samples t-tests and chi-squared tests.

Differences in peak muscle activity and CoM kinematics across groups

Differences in peak muscle activity and CoM kinematics across groups were assessed with ANOVAs with a group factor (HYA vs. Non-PD vs. PD) and with a participant factor included as a random factor nested within group. Each observation entered into ANOVA was the average of all trials of a given perturbation direction for each participant. Significant initial F tests were followed post-hoc subgroup F tests comparing 1) PD vs. Non-PD, and 2) HYA vs. older (PD or Non-PD). P values from post-hoc tests were adjusted using a Holm-Bonferroni sequential procedure.[80] Separate ANOVAs were performed for each variable for forward and backward perturbation directions.

Differences in SRM parameters across groups

Differences in SRM parameters across groups were assessed with one-way ANOVAs (HYA vs. Non-PD vs. PD). Each observation corresponded to an individual participant. Significant initial F tests were followed with Holm-Bonferroni-adjusted post-hoc independent-samples t-tests comparing: 1) PD vs. Non-PD, and, 2) HYA vs. older (PD or Non-PD). Separate batteries of ANOVA and post-hoc tests were performed for each SRM parameter.

Fall history classification

Participants were classified as having 0, 1, or ⩾2 falls in the 6 months prior to study enrollment. For older participants, fall history was obtained via self-report on the day of testing. Falls were defined as “an event that results in a person coming to rest unintentionally on the ground or another lower level,” a shortened version of an existing definition.[81,82] Young healthy participants were coded as free of falls for primary analyses. Analyses were iterated with and without young healthy participants included to evaluate sensitivity. One participant for whom fall history data were missing was excluded from analyses involving fall history.

Associations between fall history and SRM parameters

Associations between TA SRM parameter k_a' and fall history were assessed in two ways. Multivariate ANOVA assessed variation in SRM parameters with fall classification as described above. Negative binomial regression assessed association between k_a' and the number of falls over the prior 6 months among those participants for whom these data were available. Both approaches included covariates associated with fall risk: increased age, female sex, presence of PD, and presence of FOG.[10]

Associations between SRM parameters and clinical and demographic variables

We summarized associations between k_a' and clinical variables with Pearson product-moment correlation coefficients. Correlation coefficients were classified as negligible or non-negligible according to criteria proposed by Cohen.[43]

Delay differences between stabilizing and destabilizing pathways

Identified SRM delay parameters for each participant were entered into a linear mixed model with fixed effects of Pathway (TA-TA, MG-MG, and MG-TA) and PD and a random effect for participant using the lmerTest package in R software.

Tables

Table 1. Demographic and clinical characteristics of PD and age-matched Non-PD participants.

Variable	PD	Non-PD	Total
N	44	18	62
Age, y	68 ± 7	66 ± 8	68 ± 7
Sex			
Male, n (%)	23 (52)	7 (39)	30 (48)
Female, n (%)	21 (48)	11 (61)	32 (52)
Height, cm	169 ± 10	167 ± 11	169 ± 10
Weight, kg	76 ± 14	76 ± 15	77 ± 14
MoCA, /30	27.8 ± 1.5	27.2 ± 1.4	27.6 ± 1.5
PD duration, y	7.4 ± 4.8		
MDS UPDRS-III, /132	36.1 ± 13.0		
LED, mg[a]	726 ± 357		
Freezing of Gait			
Nonfreezer, n (%)	29 (66)		
Freezer, n (%)	15 (34)		
PD Phenotype			
PIGD, n (%)	27 (61)		
TD, n (%)	13 (30)		
Indet, n (%)	4 (9)		
Balance outcomes			
Mini-BESTest, /28[b,†]	22.1 ± 4.1	24.7 ± 1.4	22.8 ± 3.7
FAB, /40[c,†]	29.8 ± 5.5	33.2 ± 2.7	30.9 ± 5.0
DGI, /24[d,†]	20.4 ± 3.5	22.3 ± 1.3	21.0 ± 3.1
BBS, /56[d,†]	52.9 ± 5.5	55.0 ± 1.3	53.6 ± 3.4

Abbreviations: PD, Parkinson disease; MoCA, Montreal Cognitive Assessment; UPDRS-III, Unified Parkinson Disease Rating Scale, Part III: Motor Exam; LED, levodopa equivalent daily dose; PIGD, Postural Instability/Gait Difficulty; TD, Tremor Dominant; Indet., Indeterminate; BBS, Berg Balance Scale; FAB, Fullerton Advanced Balance Scale; DGI, Dynamic Gait Index. [a]N=41. Two patients had not yet begun pharmacotherapy and dosage information was unavailable for one patient who was prescribed carbidopa/levodopa. LED was calculated as in previous studies[29] with conversion factor of 0.6 assumed for Rytary. [b]N=28. [c]N=31. [d]N=30. Demographic information for the reference sample of young healthy participants (N=6) is provided in the main text.

Figure legends

Figure 1

Postural responses in healthy aging and in Parkinson disease. A: Schematic depiction of testing paradigm. B: Representative postural responses in healthy aging and Parkinson disease. Left: a healthy older woman (age, 69 y; no recent falls; Mini-BESTest score 23/28). Right: an older woman with PD (age, 73 y; disease duration, 20 y; 1 fall in the previous 6 months; MiniBESTest score 24/28; MDS-UPDRS-III OFF score 26/132; complaints of freezing of gait (FoG); LEDD 675 mg). In response to a backward (270°) perturbation, the center of mass (CoM) initially moves in the forward direction (*start* epoch) such that the CoM kinematic signals [displacement (*d*), velocity (*v*), acceleration (*a*)] are all opposite the direction of platform motion. When the platform decelerated (*stop* epoch), CoM acceleration quickly reversed direction to be opposite that of CoM velocity and displacement. Activity of both MG and TA muscle initiated about 100ms after the onset of perturbation in and varied in direct response to the preceding CoM kinematics signals. In both healthy and PD participants, the medial gastrocnemius (MG) activated in the *start* epoch and continued throughout the perturbation; MG is considered the agonist for restoring balance. However, in the healthy individual, there was little activation of tibialis anterior (TA) muscle in the healthy participant, but a marked burst in the TA in the *start* epoch, where TA is considered an antagonist for restoring balance. In both the healthy and PD participant, TA was activated in the *stop* epoch, where the TA is an agonist for balance correction and MG is the antagonist. Overlaid traces shown are for individual trials (5 total). Averages of all traces are shown in black.

Figure 2

Reconstruction of balance EMG with combinations of balance-correcting and nonspecific CoM feedback. A: Representative CoM feedback responses to backward support surface perturbations over healthy aging and in Parkinson disease. Left: a healthy young woman recruited from a college campus (age, 22 y). Middle: a healthy older man (age, 64 y; no recent fall history). Right: an older woman with PD (age, 62 y, disease duration, 5 y; 1 fall in the previous 6 months; MDS-UPDRS-III OFF score 55/132; no complaints of FoG; LEDD 900 mg).

Above: MG; Below: TA (reversed scale). Thin lines represent are average EMG (typically 5 trials). Thick lines represent SRM fits. Green and red shaded areas represent balance-correcting and nonspecific SRM feedback. Inset: variance accounted for (VAF) and R^2 values indicating goodness of fit. B: Feedback models for balance control. In the model formulation, MG responded to balance-correcting forward-directed kinematic signals only (green) and TA responded to balance-correcting backward-directed (green) and nonspecific forward-directed (red) kinematic feedback. C: Comparison of model parameters across groups. Individual participant data are shown as separate gray dots for each of the left and right legs. ***P<0.001, **P<0.01, *P<0.05, older vs. younger, ANOVA, post-hoc tests. †††P<0.001, PD vs. Non-PD, ANOVA, post-hoc tests.

Figure 3

Associations between TA antagonist acceleration feedback parameter $k_a{}'$ and fall history over the prior 6 months. A: Histogram of stratified fall frequency in participants with PD (blue) and without PD (brown). P value reflects χ^2 test of homogeneity. B1,2: Estimated marginal means (±SEM) describing effects of (B1) fall history classification and (B2) PD on $k_a{}'$, controlling for age, sex, and presence of FOG. P values reflect Wald χ^2 tests. C: Negative binomial regression results describing association between frequency of falls over the 6 months prior to study enrollment and $k_a{}'$, controlling for age, sex, presence of PD, and presence of FOG. Trendlines indicate expected fall frequencies as a function of $k_a{}'$ for different simulated cases: Young female (20 y), brown dotted. Older female (68 y), brown, solid. Older female (68 y) with PD but without FOG, blue, solid. Older female (68 y) with PD and FOG, blue, dashed. P value reflects a Wald χ^2 test.

Figure 4

Associations between antagonist acceleration feedback parameter $k_a{}'$ and clinical and demographic variables among participants with PD. A: Associations between SRM parameters and clinical and demographic variables. Solid lines indicate best-fit linear regressions. N=44 except as noted. Vertical lines indicate: Mini-BESTest scores <20, proposed cutoff value for 6-month fall risk in people with PD;[44] average MoCA scores for cognitively-normal people with

PD (26±3).[74] B: Pearson product-moment correlation coefficients calculated between k_a' and clinical variables. Open bars indicate univariate correlation coefficients. Closed bars indicate correlations corrected for age effects.

Figure 5

Comparison of identified SRM delay times between destabilizing and stabilizing feedback pathways. N = 62.

Author contributions

Conceptualization: McKay, Ting.

Data collection: Bong, Factor, Hackney, Lang, McKay.

Formal analysis: McKay.

Funding acquisition: Factor, McKay, Ting.

Investigation: Bong, Hackney, Lang, McKay, Ting.

Methodology: McKay, Ting.

Project administration: McKay, Ting.

Resources: McKay, Ting.

Software: McKay, Ting.

Supervision: McKay, Ting.

Visualization: McKay.

Writing – original draft: Bong, Lang, McKay.

Writing – review & editing: McKay, Factor, Hackney, Ting.

Disclosures

Funding sources and conflict of interest

This study was supported in part by National Institutes of Health grants K25HD086276, R01HD046922, R21HD075612, UL1TR002378, UL1TR000454; Department of Veterans Affairs R&D Service Career Development Awards E7108M and N0870W, The Consolidated Anti-Aging Foundation, the APDA center for Advanced Research at Emory University, and the Sartain Lanier Family Foundation. The authors report no conflict of interest related to this study.

Financial disclosures for the previous 12 months

Dr. McKay has received research funding or support from the National Institutes of Health. Dr. McKay has received consulting fees from BioCircuit Technologies. Dr. Hackney has received research funding or support from the Veteran's Health Administration, National Institutes of Health, National Science Foundation, and the Parkinson's Foundation. Dr. Factor has received research funding or support from the National Institutes of Health, the Michael J. Fox Foundation, the CHDI Foundation, Ipsen, Jazz Pharmaceuticals, Medtronic, Sunovion, Teva, US World Meds, Vaccinex Inc., and Voyager Therapeutics. Dr. Factor has received honoraria from Acadia, Adamas, Biogen, Lundbeck, Neurocrine, Prexton Therapeutics, Sunovion, and Teva. Dr. Factor has received royalties from Blackwell Futura, Demos, and Uptodate. Dr. Ting has received research funding or support from the National Institutes of Health and the National Science Foundation.

Supporting Information

Supplementary Materials S1

Additional analyses.

Supplementary Data S1, S2

Participant-level and timecourse data.

Supplementary Materials S1

Medications

Medication information was available for all participants with PD and 17/23 participants without PD. The majority (42/44) of PD patients were prescribed antiparkinsonian medications; the remaining 2 patients were early in the disease course and had not yet begun pharmacotherapy. Of those taking medications, 38/42 were on carbidopa-levodopa, either as conventional/extended release formulations or formulated as Stalevo or Rytary. Adjunctive agents included dopamine agonists (N=23), monoamine oxidase B inhibitors (N=14), entacapone (N=4), and other antiparkinsonian or anti-tremor agents including amantadine (N=5), trihexyphenidyl (N=1), apomorphine (N=1), the norepinephrine prodrug droxidopa (N=1), propranolol (N=3) and gabapentin (N=1). Two patients were taking the muscle relaxants cyclobenzaprine (N=1) and baclofen (N=1) for complications such as nocturnal cramps.

Some PD patients were taking the cholinesterase inhibitors donepezil (N=2) and memantine (N=3), and approximately one third (N=14) were taking psychiatric medications (SSRI, N=6; atypical antidepressants, N=4; SNRI, N=4, SARI, N=1). Among the Non-PD group for whom medication information was available, one person was prescribed bupropion; otherwise all neurotypical participants denied significant neurological or musculoskeletal problems. Common geriatric medications without psychiatric effects (statins, omeprazole) were frequently observed in both groups but were not abstracted.

Differences in peak muscle activity and CoM motion across groups

In addition to SRM analyses reported in the main text, gross magnitudes of CoM motion and normalized EMG magnitudes were compared between PD and Non-PD groups with independent *t*-tests (Table S1). Peak values were calculated 200, 400, and 675 ms after perturbation onset for absolute values of acceleration, velocity, and displacement, respectively in the anterior-posterior direction after averaging across replicates of each perturbation direction for each participant. Peak values for EMG were calculated during fixed windows 100-600 ms after perturbation onset.

Table S1. Comparison of gross CoM kinematics EMG activity between study groups.

Parameter	Young N=6	Non-PD N=18	PD N=44	P
Backward perturbations				
CoM kinematics				
Peak acceleration (g)	0.098±0.004	0.098±0.004	0.095±0.007	0.18
Peak velocity (cm/s)	11.9±0.5	11.3±2.5	12.3±0.9	0.07
Peak displacement (cm)	4.3±0.3	4.5±1.3	4.3±0.5	0.73
EMG activity, nu				
Peak TA, left	0.08±0.5	0.25±0.15	0.39±0.22	<0.01[*,††,‡]
Mean TA, left	0.03±0.01	0.08±0.04[a]	0.11±0.07	<0.01[*,††,‡]
Peak MG, left	0.71±0.09	0.80±0.11	0.80±0.08	0.08
Mean MG, left	0.21±0.04	0.29±0.09	0.27±0.07	0.12
Peak TA, right	0.09±0.05	0.28±0.17	0.36±0.17	<0.01[*,†]
Mean TA, right	0.03±0.01	0.10±0.07	0.13±0.07	<0.01[*,†]
Peak MG, right	0.70±0.11	0.80±0.07	0.79±0.11	0.17
Mean MG, right	0.22±0.05	0.29±0.07	0.27±0.08	0.13
Forward perturbations				
CoM kinematics				
Peak acceleration (g)	0.074±0.007	0.088±0.009	0.086±0.007	<0.01[*,††]
Peak velocity (cm/s)	11.6±0.5	11.2±2.2	12.1±1.1	0.10
Peak displacement (cm)	3.9±0.7	4.3±1.6	4.2±0.7	0.77
EMG activity, nu				
Peak TA, left	0.84±0.13	0.93±0.04	0.90±0.10	0.08
Mean TA, left	0.27±0.05	0.36±0.07	0.34±0.09	0.04[*,†]
Peak MG, left	0.33±0.18	0.37±0.13	0.32±0.16	0.51
Mean MG, left	0.10±0.05	0.14±0.06	0.12±0.06	0.25
Peak TA, right	0.84±0.09	0.90±0.05	0.88±0.08	0.28
Mean TA, right	0.27±0.08	0.34±0.07	0.34±0.08	0.14
Peak MG, right	0.39±0.31	0.33±0.15	0.40±0.23	0.47
Mean MG, right	0.12±0.11	0.12±0.05	0.14±0.07	0.48

Abbreviations: nu, normalized units; TA, tibialis anterior; MG, medial gastrocnemius. *Significant difference between groups, ANOVA. [†]P<0.05, [††]P<0.01; young vs. old, post hoc F-test, Holm-Bonferroni correction. [‡]P<0.05 PD vs. non-PD, post hoc F-test, Holm-Bonferroni correction. Results from the left leg are reported in the main text.

Recording and processing of kinetic, kinematic, and EMG data

All perturbation response data were collected and processed using procedures described previously.[10,17,35] Perturbations were applied using a custom perturbation platform (Factory Automation Systems, Atlanta, GA) driven by servo motors and controlled by industrial motion controllers. Platform acceleration was measured using a three-dimensional accelerometer directly mounted onto the platform (Analog Devices, Norwood, MA). Platform position was measured using a linear variable differential transformer (MTS Systems, Cary, NC). Ground reaction forces were captured using two six-axis commercial load cells directly mounted onto the platform (AMTI, Watertown, MA).

Analog data (platform kinematics, ground reaction forces, and EMG) were collected simultaneously and synchronized using Vicon (Oxford Metrics, Denver, CO) equipment at either 1080 or 1200 Hz depending on equipment version. Analog signals were anti-alias filtered with single pole hardware filters (500 Hz) prior to analog-to-digital conversion. Subsequent filtering was performed in software with third-order zero-lag Butterworth filters using *filtfilt.m* in Matlab. Platform signals were low-pass filtered at 30 Hz, and ground reaction forces were low-pass filtered at 100 Hz.

Kinematic marker data were collected at 120 Hz and synchronized to analog signals using Vicon hardware and software. Body segment kinematics were derived from a custom 25-marker set that included head–arms–trunk (HAT), thigh, shank, and foot segments (Vicon, Centennial, CO).[17] CoM displacement was calculated from kinematic data as a weighted sum of segmental masses.[83] CoM displacement was low-pass filtered (50 Hz) and numerically differentiated to derive the CoM velocity. CoM acceleration was then calculated as the difference between ground reaction force divided by subject mass and platform acceleration.

Surface EMG activity was collected from the following lower limb muscles: tibialis anterior (TA), medial gastrocnemius (MGAS), biceps femoris long head, rectus femoris, soleus, and vastus medialis. Only TA and MGAS were studied numerically. Silver/silver chloride disc electrodes were placed 2 cm apart at the motor point.[84] EMG data were recorded using

telemetered EMG (Konigsburg, Pasadena, CA, or Motion Lab Systems, Inc., Baton Rouge, LA) and processed offline (high-pass, 35 Hz, de-mean, rectify, low-pass, 40 Hz).[10,15,35,75]

Treatment of stepping responses

Perturbation trials that induced stepping responses or arm movement were excluded from analyses during initial processing in Vicon software. Stepping responses were identified programmatically by identifying trials in which the ground reaction force magnitude below either foot decreased below 20 N. Because of the data processing pipeline used, in which trials could be excluded at any of several stages of processing (initial processing in Vicon software via visual inspection, examination of recorded but unregistered data traces, statistical comparison of detailed numerical values), it was not feasible to systematically track exclusions. The number of trials excluded for any reason was ≈10%.

Preparation of kinematic signals for SRM Analysis

Anterior-posterior components of CoM motion were used in all analyses. We hypothesized that muscle spindles transiently encode acceleration when a muscle is stretched starting from rest, with the encoding ending abruptly as force and strain accumulate within the fiber. Although the mechanisms for such a "stiction" response are not known, it could be caused by the rapid detachment of cross-bridges.[77,78] We empirically modeled the muscle spindle stiction response by allowing acceleration encoding for a fixed time period τ after the onset of platform acceleration and deceleration. In an earlier study of young healthy individuals, values of τ were fixed at 75 ms.[17] Here, we fixed τ at 75 ms after initial perturbation acceleration and at 200 ms after initial perturbation deceleration based on preliminary investigations. During periods in which acceleration feedback was eliminated, acceleration signals were allowed to exponentially decay to zero with time constant 5 ms. This approach was taken because we reasoned that during these two periods, the agonist and antagonist muscles were lengthened sufficiently to detach cross-bridges, whereas during the middle of the perturbation response, lengthening would be insufficient.

SRM optimization

All optimizations were performed using the interior point algorithm implemented in *fmincon.m*, an implementation of that described in the literature.[85] Optimizations were performed in Matlab r2018b. In all cases, background activity levels identified from the start of recording to 50 ms after perturbation onset were removed from EMG recordings prior to SRM fitting. For balance-correcting feedback, a single optimization was performed to identify parameters k_a, k_v, k_d, and λ, with search bounds[86] described in Table S2.

For directionally nonspecific feedback, separate optimizations were initially performed to identify balance-correcting parameters k_a, k_v, k_d, and λ, and nonspecific parameters k_a', k_v', k_d', and λ'. In order to preserve initial burst peak values in reconstructions, limits on k_a' were set individually for each reconstructed EMG trace based on the ratio of peak EMG activity to peak acceleration. For each EMG trace, a parameter A was calculated as the ratio of the maximum value of normalized EMG activity calculated over a time window 150–275 ms after perturbation onset to the maximum value of the corresponding acceleration trace. The lower search bound for k_a' for that trace was then set to 90% of this value. A similar operation was employed to preserve peak values of braking activity 650-800 ms after perturbation onset by constraining k_a. After these two separate optimizations identified optimal values of parameter sets $(k_., \lambda)$ and $(k_.', \lambda')$, the feedback gains from the two parameter sets were concatenated into the initial guess for a final optimization. Lower and upper bounds for gain parameters during this search were set to ±10% of initial guess values; lower and upper bounds for delay parameters during this search were set to within ±10 ms of initial guess values. In all cases, additional parameters supplied to *fmincon.m* were as follows: *TolX,* $1e^{-9}$; *MaxFunEvals,* $1e^{5}$; *TolFun,* $1e^{-7}$. Remaining parameters were set to defaults. No manual tuning of optimization was performed.

Table S2. SRM parameter search values.

Parameter	Lower Bound	Initial Guess	Upper Bound
k_a' (nu/g)	0	10	15
k_v' (nu*s/cm)	0	0.01	0.04
k_d' (nu/cm)	0	0.01	0.04
λ' (ms)	90	140	210
k_a (nu/g)	0	10	15
k_v (nu*s/cm)	0	0.01	0.04
k_d (nu/cm)	0	0.01	0.04
λ (ms)	60	150	250

Comparison of identified SRM parameters across groups

Numerical values for identified SRM parameters for TA and MG are summarized in Tables S3 and S4, respectively. Independent omnibus and post-hoc F tests were applied to each parameter in each perturbation direction as described in the main text. Significant differences between groups for TA during backward perturbations are described in the main text. No statistically-significant differences between groups were identified for TA during forward perturbations or for MG in either perturbation direction.

Table S3. Identified SRM parameters for TA.

Parameter	Young N=6	Non-PD N=18	PD N=44
Backward perturbations			
$k_a'^{***,\dagger\dagger\dagger}$	0.8±0.4 (0.3-1.3)	1.5±0.9 (0.4-3.3)	3.0±1.8 (0.7-6.7)
k_v'	0.001±0.001 (0.000-0.004)	0.004±0.005 (0.000-0.021)	0.004±0.006 (0.000-0.035)
$k_d'^{\dagger\dagger\dagger}$	0.002±0.002 (0.000-0.005)	0.020±0.013 (0.000-0.037)	0.022±0.011 (0.001-0.037)
$\lambda'^{\dagger}$	136±28 (107-178)	189±27 (120-220)	181±18 (139-211)
$k_a^{\dagger\dagger\dagger}$	1.1±1.0 (0.3-2.9)	5.2±2.4 (1.1-10.8)	4.8±2.1 (1.0-9.1)
$k_v^{\dagger\dagger}$	0.006±0.003 (0.003-0.012)	0.011±0.007 (0.000-0.025)	0.013±0.005 (0.004-0.024)
k_d	0.014±0.007 (0.006-0.026)	0.014±0.008 (0.001-0.026)	0.018±0.008 (0.004-0.038)
$\lambda^{\dagger}$	151±13 (135-163)	129±19 (93-159)	130±20 (90-161)
Forward perturbations			
k_a	10.0±1.7 (8.0-12.0)	9.9±0.7 (8.6-11.4)	9.6±1.0 (7.0-12.4)
k_v	0.026±0.010 (0.011-0.038)	0.026±0.013 (0.000-0.040)	0.029±0.007 (0.008-0.040)
k_d	0.011±0.008 (0.000-0.020)	0.010±0.010 (0.000-0.028)	0.012±0.01 (0.000-0.040)
λ	126±12 (104-135)	144±16 (112-174)	134±12 (104-163)

Values are presented as sample mean ± sample standard deviation (sample range). ***Significant difference between Non-PD and PD groups, P<0.001. †††,††,†Significant difference young and older groups, P<0.001, P<0.01, P<0.05. Numerical values shown for backward perturbations are shown graphically in Figure 2C.

Table S4. Identified SRM parameters for MG.

Parameter	Young N=6	Non-PD N=18	PD N=44
Forward perturbations			
k_a'	1.7±2.3 (0.0-6.1)	1.0±1.2 (0.0-4.9)	1.7±1.3 (0.0-5.2)
k_v'	0.005±0.008 (0.000-0.021)	0.004±0.006 (0.000-0.020)	0.004±0.005 (0.000-0.020)
k_d'	0.014±0.011 (0.004-0.029)	0.018±0.013 (0.000-0.037)	0.015±0.010 (0.000-0.040)
λ'	170±26 (143-196)	184±26 (129-220)	175±22 (126-216)
k_a	3.2±1.7 (1.0-6.2)	4.7±2.1 (1.9-9.4)	3.8±1.8 (0.8-9.7)
k_v	0.010±0.006 (0.005-0.020)	0.008±0.007 (0.001-0.025)	0.009±0.005 (0.000-0.025)
k_d	0.016±0.012 (0.004-0.031)	0.020±0.011 (0.000-0.020)	0.022±0.009 (0.005-0.038)
λ	126±33 (90-178)	135±18 (89-165)	132±24 (79-184)
Backward perturbations			
k_a	7.9±1.0 (6.8-9.6)	7.5±1.3 (5.9-10.6)	7.6±1.2 (4.3-10.6)
k_v	0.010±0.002 (0.008-0.013)	0.015±0.006 (0.005-0.025)	0.017±0.007 (0.004-0.032)
k_d	0.009±0.008 (0.001-0.026)	0.011±0.009 (0.000-0.032)	0.009±0.008 (0.000-0.028)
λ	130±6 (122-134)	142±24 (107-192)	144±16 (103-185)

Values are presented as sample mean ± sample standard deviation (sample range). No statistically-significant differences between groups were observed.

Goodness of fit

Goodness of fit between average SRM reconstructions and average recorded EMG traces was evaluated with VAF (variance accounted for) and R^2. VAF was defined as 100•the square of Pearson's uncentered correlation coefficient[87] and was calculated as in previous studies.[15,88,89] R^2 was calculated by built-in function *regress.m*. In cases in which one or the other criterion could not be calculated due to rank deficiency, values of 0 were imputed.

Differences in goodness of fit between groups for each muscle in each perturbation direction were assessed with separate ANOVAs. Significant initial F tests were followed with Holm-Bonferroni-adjusted post-hoc independent-samples *t*-tests comparing: 1) PD vs. Non-PD, and, 2) HYA vs. older (PD or Non-PD). Identified goodness of fit values are summarized in Table S5. Very few statistically-significant differences were identified: a small decrease in VAF (1%) was noted among young compared to older participants in TA during backward perturbations and a moderate decrease in R^2 (0.09) was noted among PD compared to Non-PD in MG during forward perturbations.

Table S5. Goodness of fit values.

Parameter	All	Young	Non-PD	PD
VAF, %				
Backward perturbations				
TA*	82±6	81±7†	82±6	82±5
MG	81±7	80±3	79±11	81±5
Forward perturbations				
TA*	77±11	71±5	71±17	79±6
MG	82±7	78±6	84±5	82±7
R^2				
Backward perturbations				
TA	0.60±0.11	0.50±0.06	0.64±0.11	0.59±0.11
MG	0.53±0.12	0.52±0.05	0.49±0.17	0.54±0.11
Forward perturbations				
TA	0.63±0.13	0.59±0.08	0.59±0.19	0.65±0.09
MG*	0.44±0.13	0.38±0.09	0.51±0.12	0.42±0.13‡

Values are presented as sample mean ± sample standard deviation. *P<0.05 between groups, ANOVA. †P<0.05 between older and younger groups, ‡P<0.05 between PD and Non-PD groups, post-hoc tests.

Changes in reconstructed muscle activity after the addition of diffuse sensorimotor feedback

In order to quantify changes in fits to recorded EMG data associated with additional nonspecific CoM feedback channels in the SRM, we compared adjusted R^2 values (R^2_a) and peak reconstructed EMG levels 100-600 ms after perturbation onset before and after the addition of the new channels. These analyses were conducted using only data from the PD group, and only using data in which muscles were initially shortened by perturbations (backward for TA and forward for MG), in the interest of parsimony.

R_a^2, referred to as the adjusted coefficient of determination,[87] was calculated from R^2 values calculated by built-in function *regress.m* according to the formula:

$$R_a^2 = 1 - \frac{n-1}{n-m-1}(1-R^2)$$

where m indicates the number of explanatory variables and n indicates the number of data points. Values were calculated assuming $m = 4$ independent explanatory variables for the balance-correcting feedback pathway, $m = 8$ independent explanatory variables for the balance-correcting and nonspecific feedback pathways, and $n = 1620$ data points in all cases.

We assessed differences in R_a^2 after the addition of nonspecific feedback with paired *t*-tests. The addition of nonspecific feedback was associated with highly statistically-significant ($P<<0.001$) increases in R_a^2 for both TA during backward perturbations and MG during forward perturbations. Average increases in R_a^2 were 0.18±0.15 (range, -0.03–0.66) for TA, and 0.13±0.14 (range, -0.03–0.56) for MG.

We assessed differences in reconstructed peak muscle activity with a paired t-test for each muscle and compared the increases between TA and MG with an independent samples t-test. After the addition of additional nonspecific CoM feedback channels, reconstructed peak muscle activity 100-600 ms after perturbation onset increased significantly for both muscles ($P<0.001$). Average increases in peak muscle activity were 0.23±0.11 normalized units (range, 0.08–0.43) for TA, and 0.14±0.10 normalized units (range, 0.03–0.40), for MG. Increases in peak muscle activity were significantly higher in TA ($P<0.001$).

Associations between PD and falls

Simple associations between PD and fall history

Participants were classified as having 0, 1, or ⩾2 falls in the 6 months prior to study enrollment as described in the main text. Fall history in each group is summarized in Table S6. Simple associations between the presence of PD and fall history were assessed with chi-squared tests of homogeneity. Previous falls were significantly more prevalent among the PD group (47% vs. 12%; P=0.008; Figure 3A). Similar results were obtained if young participants were excluded from this analysis (47% vs. 17%; P=0.032).

Table S6. Retrospective 6-month fall history stratified by group.

Falls in prior 6 months	Young or Non-PD<br>N=24	PD<br>N=43
≥2	0 (0)	12 (28)
1	3 (12)	8 (19)
0	21 (88)	23 (53)

Values are presented as n (%).

Associations between SRM parameters and falls

Associations between SRM parameters and falls were assessed in two ways. First, we used multivariate ANOVAs to assess variation in SRM parameters with fall classification (0, 1, or ⩾2 falls in the prior 6 months, as described above). Second, in order to assess the strength of associations between SRM parameters and falls, we used negative binomial regression to assess associations between SRM parameters and the number of falls over the prior 6 months. In the subset of participants for whom fall frequencies were available, it ranged from 0 to 1 (Non-PD) or 180 (PD).

Primary analyses considered TA SRM parameter k_a'. For completeness, secondary analyses were performed in which the analysis was iterated for each of the remaining parameters identified during backward perturbations and shown in Figure 2. Family-wise false positive rate across these analyses was controlled with a Holm-Bonferroni sequential procedure.

Associations between SRM parameters and fall classification

Values for each identified parameter were analyzed with ANOVA with a factor for fall history classification (0, 1, or ⩾2 falls in the prior 6 months), as well as clinical and demographic covariates known to be associated with fall risk: age, female sex, presence of PD, and presence of FOG.[52,90] PD patients were classified as freezers if they scored > 1 on FOG-Q[73] item 3, indicating freezing more than once per week.[53] The age variable was transformed to z-scores prior to entry into analysis assuming mean value 68 y and standard deviation 7 y.

Compared to among participants with no fall history, $k_a{}'$ was significantly increased (115%, P<0.001, ANOVA; Figure 3B) among those with ⩾2 falls in the prior 6 months after controlling for age, sex, presence of PD, and presence of FOG. In this multivariate analysis, $k_a{}'$ was significantly increased among participants with PD (37%, P=0.042; Figure 3C) and tended to increase with age (≈8%/decade, P=0.116). No significant effects of sex (P=0.742) or presence of FOG (P=0.584) were identified. No significant differences were identified in $k_a{}'$ between participants with 1 and 0 falls over the prior 6 months.

Overall, results were very similar when the analysis was iterated with young healthy participants excluded. The highly significant effect of fall history (111%, P<0.001) was retained, the effect of PD (37%, P=0.051) was weakened slightly, and the effect of age (≈10%/decade, P=0.348) was slightly increased in magnitude.

We applied identical analyses to other SRM parameters in order to identify potential associations with fall history. Significant associations were identified for TA $k_v{}'$ (P=0.010), TA $k_d{}'$ (P=0.024), and for TA k_a (P=0.032). However, none remained statistically-significant after correction for N=11 simultaneous tests using a Holm-Bonferroni procedure.

Associations between SRM parameters and fall number

In a subset of participants (N=22, PD; N=11, Non-PD), the number of falls over the 6 months preceding study enrollment was available for analysis. Because we noted anecdotally that PD patients with extreme values of $k_a{}'$ also often reported very frequent falls, we evaluated whether this association held at the group level using negative binomial regression. We fit the following equation:

$$log\ log\ (\hat{f}) = \beta_0 + \beta_{ka'} k_a' + \beta_{age} age + \beta_{female} female + \beta_{PD} PD + \beta_{FOG} FOG$$

where $\hat{f}$ is the estimated number of falls for a given patient, which is assumed to follow a negative binomial discrete probability distribution. The regression parameters $\beta_.$ describe the intercept (β_0), and slope parameters for each of the continuous variables k_a' and age, and for each of the three dichotomous indicator variables for the presence of female sex, PD, and FOG. The age variable was standardized according to mean 68 y and standard deviation 7 y prior to entry into analysis.

The use of a negative binomial distribution was strongly supported by overdispersion in reported fall frequencies, with mean 6.9 falls/6 months and variance 875.8 falls/6 months (range 0–180). By group, sample means (variance) were 0.1 (0.1) and 12.2 (1517.7) in Non-PD and PD, respectively.

Negative binomial regression demonstrated that k_a' was highly significantly associated with increased fall frequency (P<0.001, Figure 3D). Identified regression parameters are shown in Table S7. Associations between fall frequency and k_a' remained highly statistically significant (P<0.001) when the analysis was iterated with young participants excluded.

Table S7. Negative binomial regression model parameters describing association between number of falls and SRM parameter k_a'.

Parameter	Estimate	95% CI	P Value
Intercept	-3.49	-5.49, -1.50	<0.001
k_a'	0.66	0.32, 1.00	<0.001
PD	1.24	-0.93, 3.40	0.264
Age	0.41	-0.39, 1.22	0.317
Female	1.31	-0.08, 2.70	0.064
FOG	1.07	-0.46, 2.60	0.171

Abbreviations: FOG, freezing of gait.

Within-subject variability of SRM parameters

We calculated test-retest reliability in a convenience sample of N=6 PD patients (age, 68±4 y; disease duration, 5±4 y; MDS-UPDRS-III score, 36±10) for whom repeat testing results were available ≈12 and ≈16 weeks after initial enrollment. The average time between observations was 33±5 days (range, 28-42 days), over which one would presumably expect little change in CoM control. We calculated intraclass correlation coefficient ICC (2,1) following the methodology of Shrout and Fleiss.[8]

The identified value of ICC (2,1) was 0.94 (95% CI 0.60-0.99), which is considered "excellent" according to cutoff values proposed by Koo and Li.[92] Average within-subject variability (CV) was 9.5%.

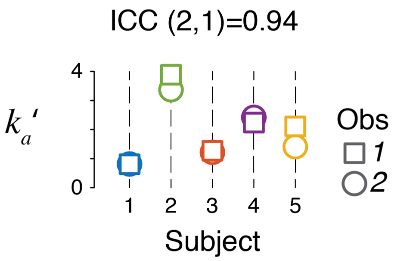

Figure S1. Within-subject variability of SRM parameter k_a'.

Associations between SRM parameters and clinical and demographic variables after controlling for age

In the main text, associations between k_a' and candidate clinical variables were summarized with Pearson product-moment correlation coefficients and classified as negligible or non-negligible according to criteria proposed by Cohen.[43] In order to control for the impact of age on identified values of k_a', we iterated correlational analyses with and without age included as a covariate and assessed the resulting changes in correlation coefficients.

In unadjusted analyses, non-negligible correlations were identified between k_a' and age, PD duration, LED, MDS-UPDRS-III total score, MDS-UPDRS-III Postural Stability score, Mini-BESTest reactive postural control score, and MoCA score, with more impaired values of k_a' associated with worse clinical measures in all cases.

In analyses adjusted for effects of age, on average correlations were attenuated in magnitude by 22 ± 29%. Only associations with PD duration, LED, and Mini-BESTest reactive postural control score remained non-negligible in adjusted models (Table S8). In these cases, more abnormal values of k_a' were associated with longer PD duration, higher amounts of pharmacotherapy, and more impaired reactive postural control.

Table S8. Single and multiple regression results describing associations between clinical variables and SRM parameter k_a'.

Variable	r	95% CI	P Value	
Univariate associations				
Age	0.18†	-0.13, 0.49	0.24	
PD duration	0.26†	-0.04, 0.56	0.09	
LED	0.19†	-0.13, 0.50	0.23	
MDS-UPDRS-III	0.10†	-0.21, 0.41	0.52	
MDS-UPDRS-III PIGD	0.04	-0.27, 0.35	0.79	
MDS-UPDRS-III Postural Stability	0.10†	-0.21, 0.41	0.50	
MDS-UPDRS-III Tremor	0.08	-0.23, 0.39	0.60	
Mini-BESTest	-0.02	-0.38, 0.35	0.93	
Mini-BESTest Reactive Postural Control	-0.12†	-0.48, 0.24	0.49	
Mini-BESTest Dynamic Gait	0.07	-0.29, 0.44	0.69	
Mini-BESTest Sensory Orientation	0.07	-0.30, 0.43	0.71	
Mini-BESTest Anticipatory Balance	-0.05	-0.42, 0.31	0.76	
MoCA	-0.12†	-0.43, 0.19	0.44	
Associations adjusted for age				Change from unadjusted
PD duration	0.23†	-0.09, 0.54	0.18	-12%
LED	0.20†	-0.11, 0.51	0.22	+5%
MDS-UPDRS-III	0.05	-0.27, 0.38	0.48	-50%
MDS-UPDRS-III PIGD	0.03	-0.28, 0.34	0.50	-25%
MDS-UPDRS-III Postural Stability	0.08	-0.24, 0.39	0.45	-20%
MDS-UPDRS-III Tremor	0.07	-0.24, 0.38	0.46	-13%
Mini-BESTest	0.00	-0.39, 0.39	0.95	-100%
Mini-BESTest Reactive Postural Control	-0.11†	-0.51, 0.28	0.78	-8%
Mini-BESTest Dynamic Gait	0.07	-0.30, 0.45	0.87	0%
Mini-BESTest Sensory Orientation	0.07	-0.30, 0.45	0.87	0%
Mini-BESTest Anticipatory Balance	-0.04	-0.44, 0.36	0.93	-20%
MoCA	-0.09	-0.40, 0.23	0.44	-25%

Abbreviations: LED, levodopa equivalent dose. †Non-negligible effect.

Examples of antagonist activity in MG

Taken together with the results earlier studies examining muscle activation throughout the leg during balance, we interpret the absence of a statistical effect of the presence of PD on MG antagonist activity as evidence that the effect of PD on MG activity is substantially more variable than the effect of PD on TA activity, rather than as evidence that PD does not affect MG activity. We were somewhat surprised that we did not find that antagonist activity in MG was abnormal in PD at the group level in this sample, given that we have previously reported excessive MG antagonist activity during balance tasks using a similar paradigm in PD patients in the ON medication state.[37] However, in a subsequent extensive examination of the activation of 6 muscles throughout the leg performed on a subset of these data, we found that the presence of PD was associated with elevated antagonist activity across generally all muscles examined, but that when muscles were considered in isolation, the effect was statistically significant only in TA. Here, although some patients exhibited strong antagonist activity in MG (see Figure S2, upper panel), others exhibited very little antagonist activity (see Figure S2, lower panel). We therefore consider the most likely explanation for these results to be that PD likely affects MG activity, although in a manner that is substantially more variable than the effect of PD on TA activity.

Why the pathophysiological processes of PD might exert a more consistent effect on TA than on MG is unknown. Many potential mechanisms could explain this type of asymmetry, although there is scarce data in humans to establish a convincing case for any particular one. Heightened rigidity in flexor rather than extensor muscles in PD – particularly in early disease stages[93] – has been noted previously, and illustrates that PD disease processes can certainly exert differential effects on particular muscle groups. In particular, Denny-Brown[94] cited the "preponderance [of rigidity] in the flexor groups" as a potential explanation for the characteristic flexed abnormal posture seen in all the limbs in terminal PD. Others, while agreeing that flexors and extensors are affected in a differential manner by PD, argue that flexed postures result primarily from extensor impairment, rather than flexor rigidity, during voluntary movements,[95] with extensor impairment potentially contributing to reduced overall strength.[96] In general, because the representations of extensor and flexor muscles are organized

in rough topographic fashion within the basal ganglia and motor cortices[97] it is reasonable that different muscle groups could be affected at in different amounts as neurodegeneration progresses along the neuraxis,[22] although the precise mechanisms of this progression remain unknown.

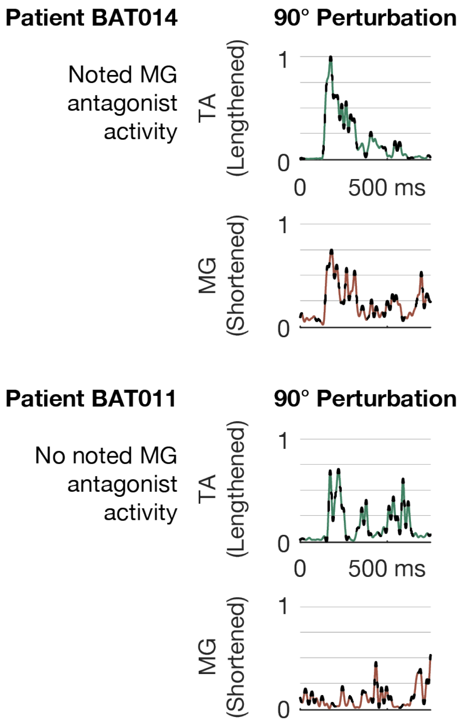

Figure S2. Examples of recorded MG antagonist activity in two participants, selected *post hoc* as exemplars of the range of MG antagonist activity seen among PD patients. Traces are averages of 3-6 replicates of 90° perturbations that initially lengthen TA and shorten MG. Muscle activity is normalized as described in the main text so that 1 corresponds to the maximum average value observed during perturbations in which the muscle is lengthened.

Additional studies in a larger number of patients with a larger number of muscles would be required to comprehensively assess the extent to which PD affects the sensorimotor control of flexors or extensors during balance, particularly because the sampling of ankle extensors is substantially more variable across studies than the sampling of ankle flexors. Here, for consistency with previous studies using the SRM approach in young healthy individuals, we considered TA and MG. However, while previous studies using perturbation paradigms to assess

balance in PD have almost uniformly recorded TA, the sampling of ankle extensors has been much more variable, precluding comparing results across studies. Of three earlier studies with methodological similarities to the testing paradigm here, the first recorded MG, but only considered backward perturbations of the support surface, so the antagonist activity of MG was never observed.[13] The others[11,12] considered perturbation directions throughout the horizontal plane, but reported soleus (SOL) rather than MG.

Simple association between SRM parameters and PD duration

A scatterplot of SRM parameter k_a' vs. PD duration is presented in Figure S3. Values are adjusted for linear effects of age. Separate best-fit regression lines are presented for PD participants ⩾5 years duration and <5 years duration. Participants with higher values of k_a' tend to be beyond 5 years PD duration. Not all participants with PD exhibit high values of k_a', but all participants with high values of k_a' have PD. There is evidence from visual inspection that the relationship between k_a' and PD duration has a nonlinearity at approximately 5 years, with a stronger and steeper relationship in the earlier period.

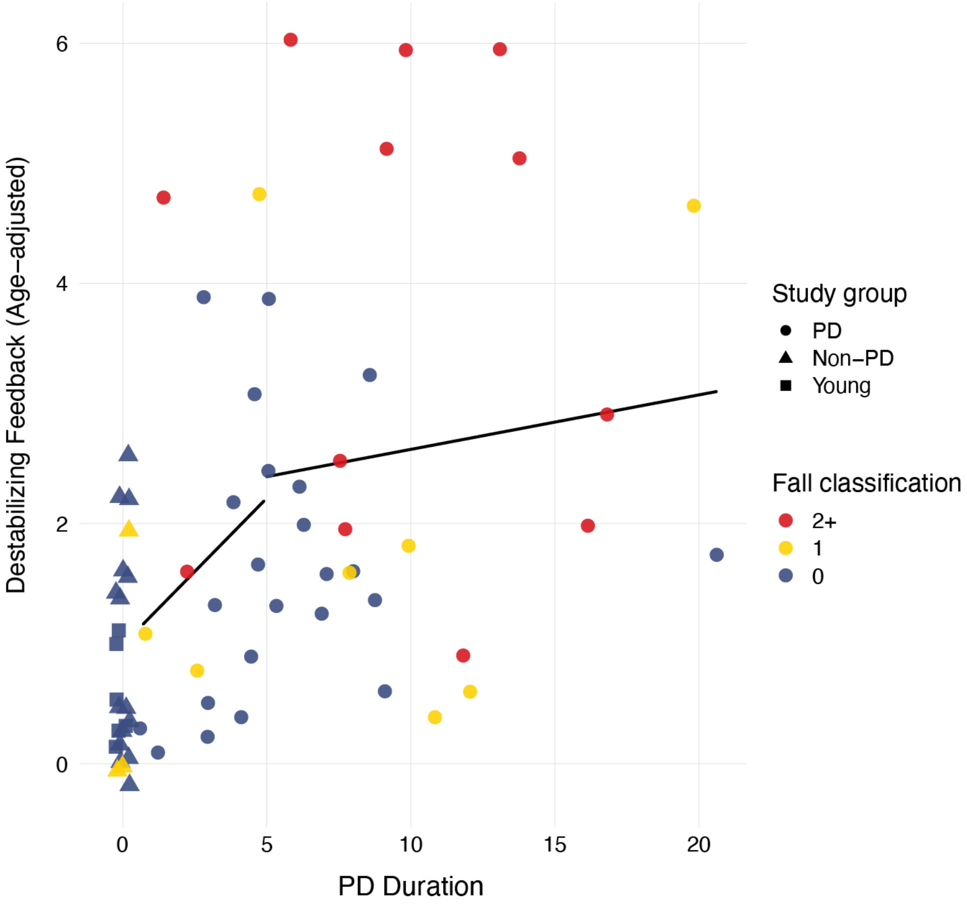

Figure S3. Comparison of k_a' and PD duration. Separate best-fit lines are presented for duration < and ⩾5 years. A small amount of horizontal jitter has been added to aid in visualization.

Simple association between SRM parameters and freezing of gait

In primary analyses of associations between k_a' and falls, presence of freezing of gait (FOG) was controlled for as a dichotomized variable, as in previous studies.[52,53] The choice of using a dichotomized measurement for FOG in primary analyses was due to the limited validity of self-reported instruments for capturing moderate or small changes in FOG severity.[98,99]

In order to more fully examine potential associations between k_a' and FOG, we performed a secondary analysis post-hoc on the dataset of k_a' and an estimate of FOG severity, FOG-Q total score. These data were available for N=42 PD patients. Overall, Pearson's associations were non-negligible, r=0.18 (P=0.25) and r=0.14 (P=0.37) before and after adjustment for age, respectively.

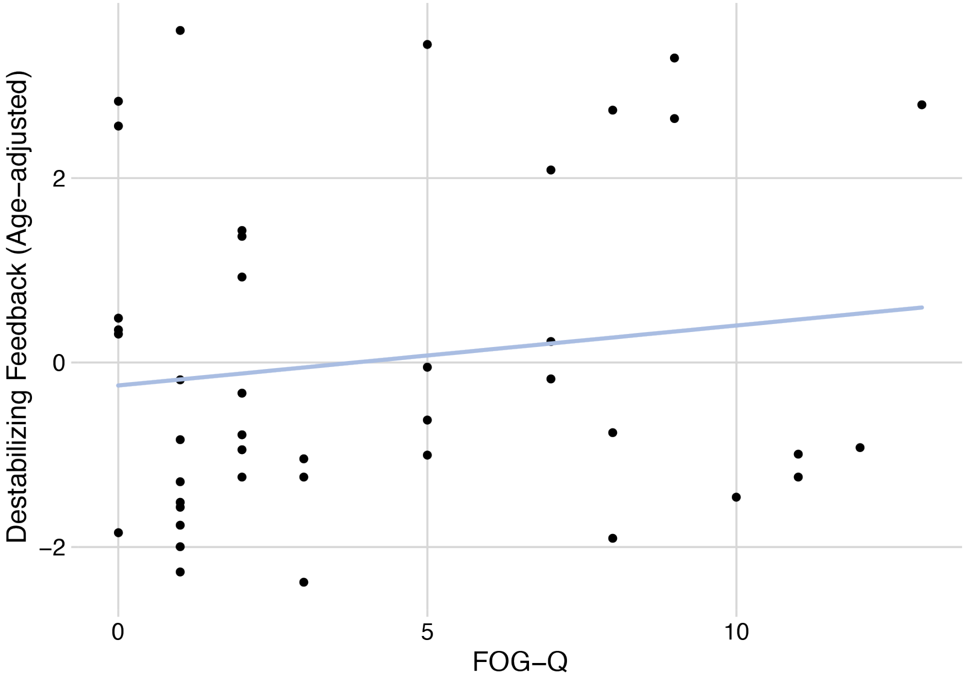

Figure S4. Comparison of k_a' (adjusted for age) and FOG-Q score.

Variation of SRM with PD phenotype

Based on earlier analyses of overall muscle activity levels[10] we did not expect large differences between PD patients with TD vs. PIGD vs. Indeterminate. We verified this with a one-way

ANOVA (PIGD vs. TD vs. Indeterminate phenotype) applied post-hoc that identified no variation in average k_a' level across groups (P=0.94).

References

1. Macpherson, J. M. & Horak, F. B. Posture. in *Principles of Neural Science* (eds. Kandel, E. R., Schwartz, J. H., Jessell, T. M., Siegelbaum, S. A. & Hudspeth, A. J.) 935–959 (McGraw-Hill, 2013).
2. Lockhart, D. B. & Ting, L. H. Optimal sensorimotor transformations for balance. *Nat Neurosci* **10**, 1329–1336 (2007).
3. Bloem, B. R., Grimbergen, Y. A. M., Cramer, M., Willemsen, M. & Zwinderman, A. H. Prospective assessment of falls in Parkinson's disease. *J Neurol* **248**, 950–958 (2001).
4. Ivanenko, Y. & Gurfinkel, V. S. Human Postural Control. *Front Neurosci* **12**, 171 (2018).
5. Winter, D. A. Human balance and posture control during standing and walking. *Gait Posture* **3**, 193–214 (1995).
6. Manckoundia, P., Mourey, F., Perennou, D. & Pfitzenmeyer, P. Backward disequilibrium in elderly subjects. *Clin Interv Aging* **3**, 667–72 (2008).
7. Weaver, T. B., Robinovitch, S. N., Laing, A. C. & Yang, Y. Falls and Parkinson's Disease: Evidence from Video Recordings of Actual Fall Events. *J. Am. Geriatr. Soc.* **64**, 96–101 (2016).
8. Angel, R. W. & Lewitt, P. A. Unloading and shortening reactions in Parkinson's disease. *J Neurol Neurosurg Psychiatry* **41**, 919–23 (1978).
9. Hallett, M. & Khoshbin, S. A physiological mechanism of bradykinesia. *Brain* **103**, 301–14 (1980).

10. Lang, K., Hackney, M., Ting, L. & McKay, J. Antagonist muscle activity during reactive balance responses is elevated in Parkinson's disease and in balance impairment. *PLoS One* **14**, e0211137 (2019).

11. Dimitrova, D., Horak, F. B. & Nutt, J. G. Postural Muscle Responses to Multidirectional Translations in Patients With Parkinson's Disease. *J Neurophysiol* **91**, 489–501 (2004).

12. Carpenter, M. G., Allum, J. H., Honegger, F., Adkin, A. L. & Bloem, B. R. Postural abnormalities to multidirectional stance perturbations in Parkinson's disease. *J Neurol Neurosurg Psychiatry* **75**, 1245–54 (2004).

13. Horak, F. B., Frank, J. & Nutt, J. Effects of dopamine on postural control in parkinsonian subjects: scaling, set, and tone. *J. Neurophysiol.* **75**, 2380–2396 (1996).

14. St George, R. J. *et al.* The effects of subthalamic and pallidal deep brain stimulation on postural responses in patients with Parkinson disease. *J Neurosurg* **116**, 1347–56 (2012).

15. Safavynia, S. A. & Ting, L. H. Long-latency muscle activity reflects continuous, delayed sensorimotor feedback of task-level and not joint-level error. *J Neurophysiol* **110**, 1278–90 (2013).

16. Welch, T. D. & Ting, L. H. Mechanisms of motor adaptation in reactive balance control. *PLoS One* **9**, e96440 (2014).

17. Welch, T. D. J. & Ting, L. H. A Feedback Model Explains the Differential Scaling of Human Postural Responses to Perturbation Acceleration and Velocity. *J Neurophysiol* **101**, 3294–3309 (2009).

18. Ting, L. H. & Macpherson, J. M. Ratio of shear to load ground-reaction force may underlie the directional tuning of the automatic postural response to rotation and translation. *J Neurophysiol* **92**, 808–23 (2004).

19. Macpherson, J. M. Strategies that simplify the control of quadrupedal stance. II. Electromyographic activity. *J. Neurophysiol.* **60**, 218–31 (1988).

20. Honeycutt, C. Mechanisms underlying muscle recruitment in response to postural perturbations. (Georgia Institute of Technology/ Emory University, 2009).

21. Honeycutt, C. F., Gottschall, J. S. & Nichols, T. R. Electromyographic Responses From the Hindlimb Muscles of the Decerebrate Cat to Horizontal Support Surface Perturbations. *J Neurophysiol* **101**, 2751–2761 (2009).

22. Braak, H. *et al.* Staging of brain pathology related to sporadic Parkinson's disease. *Neurobiol Aging* **24**, 197–211 (2003).

23. Blum, K. P. *et al. Diverse muscle spindle firing properties emerge from multiscale muscle mechanics*. http://biorxiv.org/lookup/doi/10.1101/858209 (2019) doi:10.1101/858209.

24. De Luca, C. J. & Mambrito, B. Voluntary control of motor units in human antagonist muscles: coactivation and reciprocal activation. *J Neurophysiol* **58**, 525–42 (1987).

25. DeLong, M. R. Primate models of movement disorders of basal ganglia origin. *Trends Neurosci.* **13**, 281–285 (1990).

26. Pasquereau, B., DeLong, M. R. & Turner, R. S. Primary motor cortex of the parkinsonian monkey: altered encoding of active movement. *Brain* **139**, 127–43 (2016).

27. Robichaud, J. A. *et al.* Variability of EMG patterns: a potential neurophysiological marker of Parkinson's disease? *Clin Neurophysiol* **120**, 390–7 (2009).

28. Stebbins, G. T. *et al.* How to identify tremor dominant and postural instability/gait difficulty groups with the movement disorder society unified Parkinson's disease rating scale: comparison with the unified Parkinson's disease rating scale. *Mov Disord* **28**, 668–70 (2013).

29. Tomlinson, C. L. *et al.* Systematic review of levodopa dose equivalency reporting in Parkinson's disease. *Mov Disord* **25**, 2649–53 (2010).

30. Nasreddine, Z. S. *et al.* The Montreal Cognitive Assessment, MoCA: a brief screening tool for mild cognitive impairment. *J Am Geriatr Soc* **53**, 695–9 (2005).

31. Nazem, S. *et al.* Montreal cognitive assessment performance in patients with Parkinson's disease with 'normal' global cognition according to mini-mental state examination score. *J Am Geriatr Soc* **57**, 304–8 (2009).

32. Schlenstedt, C. *et al.* Comparison of the Fullerton Advanced Balance Scale, Mini-BESTest, and Berg Balance Scale to Predict Falls in Parkinson Disease. *Phys Ther* **96**, 494–501 (2016).

33. Shumway-Cook, A. & Woollacott, M. H. *Motor Control: Theory and Practical Applications*. (Williams & Wilkins, 1995).

34. Berg, K., Wood-Dauphinee, S. & Williams, J. The Balance Scale: reliability assessment with elderly residents and patients with an acute stroke. *Scand J Rehabil Med* **27**, 27–36 (1995).

35. McKay, J. L., Ting, L. H. & Hackney, M. E. Balance, body motion, and muscle activity after high-volume short-term dance-based rehabilitation in persons with Parkinson disease: a pilot study. *J Neurol Phys Ther* **40**, 257–68 (2016).

36. Bong, S. M., McKay, J. L., Factor, S. A. & Ting, L. H. Perception of whole-body motion during balance perturbations is impaired in Parkinson's disease and is associated with balance impairment. *Gait Posture* **76**, 44–50 (2020).

37. Allen, J. L., McKay, J. L., Sawers, A., Hackney, M. E. & Ting, L. H. Increased neuromuscular consistency in gait and balance after partnered, dance-based rehabilitation in Parkinson's disease. *J Neurophysiol* **118**, 363–373 (2017).

38. Gardner, W., Mulvey, E. P. & Shaw, E. C. Regression analyses of counts and rates: Poisson, overdispersed Poisson, and negative binomial models. *Psychol Bull* **118**, 392–404 (1995).

39. Gill, D. P., Zou, G. Y., Jones, G. R. & Speechley, M. Comparison of regression models for the analysis of fall risk factors in older veterans. *Ann. Epidemiol.* **19**, 523–530 (2009).

40. Simpson, L. A., Miller, W. C. & Eng, J. J. Effect of Stroke on Fall Rate, Location and Predictors: A Prospective Comparison of Older Adults with and without Stroke. *PLoS ONE* **6**, e19431 (2011).

41. Ullah, S., Finch, C. F. & Day, L. Statistical modelling for falls count data. *Accid. Anal. Prev.* **42**, 384–392 (2010).

42. Duncan, R. P. *et al.* Comparative utility of the BESTest, mini-BESTest, and brief-BESTest for predicting falls in individuals with Parkinson disease: a cohort study. *Phys Ther* **93**, 542–50 (2013).

43. Cohen, J. A power primer. *Psychol Bull* **112**, 155–9 (1992).

44. Duncan, R. P. *et al.* Accuracy of fall prediction in Parkinson disease: six-month and 12-month prospective analyses. *Park. Dis.* **2012**, 237673 (2012).

45. Park, J. H., Kang, Y. J. & Horak, F. B. What Is Wrong with Balance in Parkinson's Disease? *J Mov Disord* **8**, 109–14 (2015).

46. Bloxham, C. A., Dick, D. J. & Moore, M. Reaction times and attention in Parkinson's disease. *J. Neurol. Neurosurg. Psychiatry* **50**, 1178–1183 (1987).

47. Kwon, D.-Y. *et al.* Quantitative Electromyographic Analysis of Reaction Time to External Auditory Stimuli in *Drug-Naïve* Parkinson's Disease. *Park. Dis.* **2014**, 1–8 (2014).

48. Evarts, E. V., Teravainen, H. & Calne, D. B. Reaction time in Parkinson's disease. *Brain* **104**, 167–86 (1981).

49. Chvatal, S. A. & Ting, L. H. Common muscle synergies for balance and walking. *Front. Comput. Neurosci.* **7**, (2013).

50. McKay, J. L. *et al.* Freezing of Gait can persist after an acute levodopa challenge in Parkinson's disease. *Npj Park. Dis.* **5**, 25 (2019).

51. Snijders, A. H. *et al.* Physiology of freezing of gait. *Ann Neurol* **80**, 644–659 (2016).

52. McKay, J. L., Hackney, M. E., Factor, S. A. & Ting, L. H. Lower Limb Rigidity Is Associated with Frequent Falls in Parkinson's Disease. *Mov Disord Clin Pr.* **6**, 446–451 (2019).

53. McKay, J. L., Lang, K. C., Ting, L. H. & Hackney, M. E. Impaired set shifting is associated with previous falls in individuals with and without Parkinson's disease. *Gait Posture* **62**, 220–226 (2018).

54. Kesar, T. M. *et al.* Agonist-Antagonist Coactivation Enhances Corticomotor Excitability of Ankle Muscles. *Neural Plast* **2019**, 5190671 (2019).

55. Crone, C. & Nielsen, J. Central control of disynaptic reciprocal inhibition in humans. *Acta Physiol Scand* **152**, 351–63 (1994).

56. Finley, J. M., Dhaher, Y. Y. & Perreault, E. J. Acceleration dependence and task-specific modulation of short- and medium-latency reflexes in the ankle extensors. *Physiol Rep* **1**, e00051 (2013).

57. Zaback, M., Adkin, A. L. & Carpenter, M. G. Adaptation of emotional state and standing balance parameters following repeated exposure to height-induced postural threat. *Sci Rep* **9**, 12449 (2019).

58. Seki, K. & Fetz, E. E. Gating of Sensory Input at Spinal and Cortical Levels during Preparation and Execution of Voluntary Movement. *J. Neurosci.* **32**, 890–902 (2012).

59. Riederer, P. & Wuketich, S. Time course of nigrostriatal degeneration in parkinson's disease. A detailed study of influential factors in human brain amine analysis. *J Neural Transm* **38**, 277–301 (1976).

60. Willard, A. M., Bouchard, R. S. & Gittis, A. H. Differential degradation of motor deficits during gradual dopamine depletion with 6-hydroxydopamine in mice. *Neuroscience* **301**, 254–67 (2015).

61. Bohnen, N. I. *et al.* Cholinergic system changes of falls and freezing of gait in Parkinson's disease. *Ann Neurol* **85**, 538–549 (2019).

62. Bohnen, N. I. *et al.* History of falls in Parkinson disease is associated with reduced cholinergic activity. *Neurology* **73**, 1670–6 (2009).

63. Hornykiewicz, O. & Kish, S. J. Biochemical pathophysiology of Parkinson's disease. *Adv Neurol* **45**, 19–34 (1987).

64. Petersen, N., Christensen, L. O., Morita, H., Sinkjaer, T. & Nielsen, J. Evidence that a transcortical pathway contributes to stretch reflexes in the tibialis anterior muscle in man. *J Physiol* **512 (Pt 1)**, 267–76 (1998).

65. Mackel, R., Iriki, A. & Brink, E. E. Spinal input to thalamic VL neurons: evidence for direct spinothalamic effects. *J Neurophysiol* **67**, 132–44 (1992).

66. Langley, J., Huddleston, D. E., Sedlacik, J., Boelmans, K. & Hu, X. P. Parkinson's disease-related increase of T2*-weighted hypointensity in substantia nigra pars compacta: SNpc Hypointensity. *Mov. Disord.* **32**, 441–449 (2017).

67. Drucker, J. H. *et al.* Internally Guided Lower Limb Movement Recruits Compensatory Cerebellar Activity in People With Parkinson's Disease. *Front. Neurol.* **10**, 537 (2019).

68. Foreman, K. B., Addison, O., Kim, H. S. & Dibble, L. E. Testing balance and fall risk in persons with Parkinson disease, an argument for ecologically valid testing. *Park. Relat Disord* **17**, 166–71 (2011).

69. Hoskovcová, M. *et al.* Predicting Falls in Parkinson Disease: What Is the Value of Instrumented Testing in OFF Medication State? *PloS One* **10**, e0139849 (2015).

70. Curtze, C., Nutt, J. G., Carlson-Kuhta, P., Mancini, M. & Horak, F. B. Levodopa Is a Double-Edged Sword for Balance and Gait in People With Parkinson's Disease: Levodopa is a Double-Edged Sword. *Mov. Disord.* **30**, 1361–1370 (2015).

71. Goetz, C. G. *et al.* The MDS-sponsored Revision of the Unified Parkinson's Disease Rating Scale. 33 (2019).

72. Goetz, C. G., Stebbins, G. T. & Tilley, B. C. Calibration of unified Parkinson's disease rating scale scores to Movement Disorder Society-unified Parkinson's disease rating scale scores. *Mov Disord* **27**, 1239–42 (2012).

73. Giladi, N. *et al.* Construction of freezing of gait questionnaire for patients with Parkinsonism. *Park. Relat D* **6**, 165–170 (2000).

74. Hoops, S. *et al.* Validity of the MoCA and MMSE in the detection of MCI and dementia in Parkinson disease. *Neurology* **73**, 1738–45 (2009).

75. Chvatal, S. A. & Ting, L. H. Voluntary and Reactive Recruitment of Locomotor Muscle Synergies during Perturbed Walking. *J. Neurosci.* **32**, 12237–12250 (2012).
76. Blum, K. P., D'Incamps, B. L., Zytnicki, D. & Ting, L. H. Force encoding in muscle spindles during stretch of passive muscle. *PLoS Comp Biol* **in press**, (2017).
77. Getz, E. B., Cooke, R. & Lehman, S. L. Phase transition in force during ramp stretches of skeletal muscle. *Biophys J* **75**, 2971–83 (1998).
78. Henatsch, H. D. [Pro and contra on the acceleration sensitivity of muscle spindles]. *Bull Schweiz Akad Med Wiss* **27**, 266–81 (1971).
79. Rice, W. R. Analyzing Tables of Statistical Tests. *Evolution* **43**, 223–225 (1989).
80. Holm, S. A Simple Sequentially Rejective Multiple Test Procedure. *Scand. J. Stat.* **6**, 65–70 (1979).
81. Stack, E. & Ashburn, A. Fall events described by people with Parkinson's disease: implications for clinical interviewing and the research agenda. *Physiother Res Int* **4**, 190–200 (1999).
82. Clark, R. D., Lord, S. R. & Webster, I. W. Clinical parameters associated with falls in an elderly population. *Gerontology* **39**, 117–23 (1993).
83. Winter, D. A. *Biomechanics and Motor Control of Human Movement*. (John Wiley & Sons, 2005).
84. Basmajian, J. V. & Blumenstein, R. *Electrode Placement in EMG Biofeedback*. (Williams & Wilkins, 1980).

85. Waltz, R. A., Morales, J. L., Nocedal, J. & Orban, D. An interior algorithm for nonlinear optimization that combines line search and trust region steps. *Math. Program.* **107**, 391–408 (2006).

86. Welch, T. D. J. A feedback model for the evaluation of the adaptive changes to temporal muscle activation patterns following postural disturbance. (Emory University and Georgia Tech, 2008).

87. Zar, J. *Biostatistical Analysis*. (Prentice-Hall, 1999).

88. Torres-Oviedo, G., Macpherson, J. M. & Ting, L. H. Muscle synergy organization is robust across a variety of postural perturbations. *J. Neurophysiol.* **96**, 1530–1546 (2006).

89. Chvatal, S. A., Macpherson, J. M., Torres-Oviedo, G. & Ting, L. H. Absence of postural muscle synergies for balance after spinal cord transection. *J Neurophysiol* **110**, 1301–10 (2013).

90. Paul, S. S. *et al.* Three simple clinical tests to accurately predict falls in people with Parkinson's disease. *Mov Disord* **28**, 655–62 (2013).

91. Shrout, P. E. & Fleiss, J. L. Intraclass correlations: uses in assessing rater reliability. *Psychol Bull* **86**, 420–8 (1979).

92. Koo, T. K. & Li, M. Y. A Guideline of Selecting and Reporting Intraclass Correlation Coefficients for Reliability Research. *J Chiropr Med* **15**, 155–63 (2016).

93. Rodriguez-Oroz, M. C. *et al.* Initial clinical manifestations of Parkinson's disease: features and pathophysiological mechanisms. *Lancet Neurol* **8**, 1128–39 (2009).

94. Denny-Brown, D. Diseases of the basal ganglia. Their relation to disorders of movement. *Lancet* **2**, 1099–105 (1960).

95. Robichaud, J. A., Pfann, K. D., Comella, C. L., Brandabur, M. & Corcos, D. M. Greater impairment of extension movements as compared to flexion movements in Parkinson's disease. *Exp Brain Res* **156**, 240–54 (2004).

96. Inkster, L. M., Eng, J. J., MacIntyre, D. L. & Stoessl, A. J. Leg muscle strength is reduced in Parkinson's disease and relates to the ability to rise from a chair. *Mov Disord* **18**, 157–62 (2003).

97. Nambu, A. Somatotopic organization of the primate Basal Ganglia. *Front Neuroanat* **5**, 26 (2011).

98. Hulzinga, F. *et al.* The New Freezing of Gait Questionnaire: Unsuitable as an Outcome in Clinical Trials? *Mov. Disord. Clin. Pract.* mdc3.12893 (2020) doi:10.1002/mdc3.12893.

99. Snijders, A. H., Haaxma, C. A., Hagen, Y. J., Munneke, M. & Bloem, B. R. Freezer or non-freezer: Clinical assessment of freezing of gait. *Parkinsonism Relat. Disord.* **18**, 149–154 (2012).
